# Effects of a Synbiotic on Plasma Immune Activity Markers and Short-Chain Fatty Acids in Children and Adults with ADHD—A Randomized Controlled Trial

**DOI:** 10.3390/nu15051293

**Published:** 2023-03-06

**Authors:** Liu L. Yang, Miranda Stiernborg, Elin Skott, Jingjing Xu, Yujiao Wu, Rikard Landberg, Samsul Arefin, Karolina Kublickiene, Vincent Millischer, Ida A. K. Nilsson, Martin Schalling, MaiBritt Giacobini, Catharina Lavebratt

**Affiliations:** 1 Department of Molecular Medicine and Surgery, Karolinska Institutet, 171 76 Stockholm, Sweden; 2 Center for Molecular Medicine, Karolinska University Hospital Solna, 171 76 Stockholm, Sweden; 3 Department of Neurology, Union Hospital, Tongji Medical College, Huazhong University of Science and Technology, Wuhan 430074, China; 4 PRIMA Child and Adult Psychiatry Stockholm AB, 163 74 Rinkeby, Sweden; 5 Department of Medical Biochemistry and Biophysics, Karolinska Institutet, 171 76 Stockholm, Sweden; 6 Department of Biology and Biological Engineering, Chalmers University of Technology, 412 96 Gothenburg, Sweden; 7 Division of Renal Medicine, Department of Clinical Science, Intervention & Technology, Karolinska Institutet, 141 52 Huddinge, Sweden; 8 Department of Psychiatry and Psychotherapy, Medical University of Vienna, 1090 Vienna, Austria

**Keywords:** acetic acid, propionic acid, IL-12, ICAM-1, VCAM-1, psychostimulants

## Abstract

Synbiotic 2000, a pre + probiotic, reduced comorbid autistic traits and emotion dysregulation in attention deficit hyperactivity disorder (ADHD) patients. Immune activity and bacteria-derived short-chain fatty acids (SCFAs) are microbiota–gut–brain axis mediators. The aim was to investigate Synbiotic 2000 effects on plasma levels of immune activity markers and SCFAs in children and adults with ADHD. ADHD patients (n = 182) completed the 9-week intervention with Synbiotic 2000 or placebo and 156 provided blood samples. Healthy adult controls (n = 57) provided baseline samples. At baseline, adults with ADHD had higher pro-inflammatory sICAM-1 and sVCAM-1 and lower SCFA levels than controls. Children with ADHD had higher baseline sICAM-1, sVCAM-1, IL-12/IL-23p40, IL-2Rα, and lower formic, acetic, and propionic acid levels than adults with ADHD. sICAM-1, sVCAM-1, and propionic acid levels were more abnormal in children on medication. Synbiotic 2000, compared to placebo, reduced IL-12/IL-23p40 and sICAM-1 and increased propionic acid levels in children on medication. SCFAs correlated negatively with sICAM-1 and sVCAM-1. Preliminary human aortic smooth-muscle-cell experiments indicated that SCFAs protected against IL-1β-induced ICAM-1 expression. These findings suggest that treatment with Synbiotic 2000 reduces IL12/IL-23p40 and sICAM-1 and increases propionic acid levels in children with ADHD. Propionic acid, together with formic and acetic acid, may contribute to the lowering of the higher-than-normal sICAM-1 levels.

## 1. Introduction

Attention deficit hyperactivity disorder (ADHD) is a common childhood-onset neurodevelopmental psychiatric disorder with about 5% worldwide prevalence among children and adolescents and 3% in adults [1]. The core symptoms of the disorder are inattention and hyperactivity/impulsivity, which lead to functional impairments in life at school, work, home, and/or social activity [2]. ADHD is markedly heterogenic regarding clinical features and likely also in etiological and pathophysiological aspects. Around 75–80% of cases have a comorbid psychiatric condition (e.g., mood disorder, anxiety disorder, learning disorder, tic disorder, or autism spectrum disorder (ASD) [2,3]. The co-occurrence of ADHD with immune-mediated conditions, such as asthma and celiac disease, proposes that there is an altered immune response in ADHD [3,4]. Additionally, prenatal exposure to inflammation has been suggested to increase the risk for ADHD [3]. Current treatments available for ADHD, including medications and behavioral therapies, are to manage the symptoms. Micronutrients and vitamin D have been weakly supported for the treatment of ADHD [5].

Preclinical studies from germ-free and antibiotic drug-treated mouse models have shown that the absence or alteration of normal gut microbiota early in life has significant effects on immune activity [6], stress responsiveness, and behaviors resembling traits of hyperactivity, depression, anxiety, autism, and obsessive-compulsive behaviors [7,8,9,10,11,12,13]. The gut microbiome in ADHD has been reported to be different compared to that in healthy controls, although no specific ADHD-associated gut bacterial taxa have been confirmed [14,15,16,17,18,19,20,21,22]. Transfer of fecal microbiota from ADHD patients to mice reduced the murine brain structural integrity and functional connectivity and increased anxiety-like behavior. Thus, an altered microbiota state in ADHD may contribute to some behaviors in ADHD through the microbiota–gut–brain axis [23]. Likewise, fecal microbiota transfer from patients with depression, autism, or schizophrenia to rodent models induced corresponding disease-like behaviors [24,25,26]. We have shown that early life antibiotic exposure was associated with an increased risk of several psychiatric disorders, including ADHD [27]. Interventional strategies have provided treatment potential. Placebo-controlled clinical trials of probiotic interventions indicated positive effects on reducing symptoms of depression, anxiety, autism, and emotion-related behaviors [28,29,30,31]. Our randomized placebo-controlled trial of Synbiotic 2000, containing 3 lactic acid bacilli and 4 dietary fibers, also showed positive effects on autistic symptoms and emotion regulation in ADHD patients who, at baseline, had higher plasma levels of vascular inflammation markers [31]. However, the mechanisms behind the intervention effects have yet to be determined.

Short-chain fatty acids (SCFAs) have been proposed to be messengers for microbiota–gut–brain communication. They are fatty acids with less than six carbon atoms, which are mainly generated by anaerobic colonic bacteria via fermentation of dietary fibers or branched-chain amino acids [32]. The most abundant SCFAs in stool and body fluids are formic acids, acetic acid, propionic acid, and butyric acid (Human Metabolome Database, http://www.hmdb.ca, accessed on 15 September 2022). Succinic acid is an intermediate metabolite in the fermentation towards propionic acid. SCFAs are multifunctional molecules, being not only an essential energy source for local intestinal cells [33,34] but also influencing barrier function, neurotransmitter release, microglial maturation and activation, neural proliferation, mitochondrial function, immune-modulation, and anti-inflammatory processes [11,34,35,36,37,38,39,40,41]. These effects are likely mediated by the SCFA receptors (GPR41/GPR43/GPR109a) or by the histone deacetylase (HDAC) inhibitory activity epigenetically regulating gene expression [42]. Succinic acid also contributes to an adequate immune response and the regulation of blood pressure and thermogenesis [43].

Immune activity is also considered to be an important mediator in the microbiota–gut–brain axis [44,45,46]. In the last two decades, studies have revealed associations between immune activation and several neuropsychiatric disorders, especially by measuring circulating inflammatory markers [47]. The peripheral immune activity markers C-reactive protein (CRP), interleukin (IL)-1β, IL-6, IL-10, IL-18, transforming growth factor (TGF)-β1, tumor neurosis factor (TNF)-α, monocyte chemoattractant protein 1 (MCP-1), eotaxin-1, and sIL-2R were reported associated with mood disorders, schizophrenia (SZ) and ASD in meta-analyses [48,49,50] suggesting immune activation in the pathophysiology of these disorders [51,52,53]. However, only a few studies focused on ADHD, and the sample sizes were small [54,55]. A recent meta-analysis of children and adults with ADHD analyzed pro-inflammatory CRP, IL-1β, IL-6, IFN-α, TNF-α, and anti-inflammatory IL-10 and reported increased IL-6 and reduced TNF-α in children with ADHD compared to controls, while the other markers were not significantly different in ADHD patients [56]. Levels of IL-12/IL-23p40 in cerebrospinal fluid (CSF) were elevated in patients with SZ [57]. Intercellular adhesion molecule 1 (ICAM-1) has been recognized in psychiatric disorders because of its putative role in neuroinflammation and the blood–brain barrier (BBB) function [58]. Higher plasma levels of its soluble form, sICAM-1, were found in ADHD among children [59]. Elevated levels of soluble or membrane-bound ICAM-1 and VCAM-1 levels have been reported in the CSF or brains of individuals with schizophrenia, unipolar or bipolar depression [60,61,62]. Moreover, chronic oral exposure to methylphenidate, a commonly used ADHD medication, has at high clinically relevant doses been shown to cause microglia activation and neuroinflammation in the cerebral cortex, hippocampus, thalamus, and basal ganglia [63], and BBB hyperpermeability [64] in rodent brain. Likewise, the use of dexamphetamine has been reported to induce neuroinflammation in rodents [65,66]. Notably, children with psychostimulant medication for ADHD had higher plasma levels of sICAM-1 and sVCAM-1 than those without this medication [67]. A recent large epidemiological study found that ADHD is a risk factor for cardiovascular disease [68] in which ICAM-1 and VCAM-1 are known to often be upregulated [69].

The aim of this study was to explore the effects of Synbiotic 2000 on concentrations of plasma immune activity markers and SCFAs in ADHD. These analyte concentrations constitute secondary outcome measures in the placebo-controlled randomized trial ISRCTN57795429 (https://doi.org/10.1186/ISRCTN57795429).

## 2. Materials and Methods

### 2.1. Participants

All participants in this study, including ADHD patients and healthy controls, were recruited through a double-blind randomized controlled trial (ISRCTN57795429) of Synbiotic 2000 intervention performed between January 2016 and June 2018 at psychiatric clinics in Stockholm, Sweden, as previously described [31]. Patients included (n = 248) all had a prior ADHD diagnosis (based on criteria from ICD-10 or DSM-5), were 5–55 years old, and were, if treated, on a stable pharmacological treatment (the last four weeks before recruitment), were not on antibiotic treatment (the last six weeks) and did not have a gastrointestinal (GI)-diagnosis (except irritable bowel syndrome), diabetes or celiac disease. In parallel, adult healthy individuals without an ADHD diagnosis (n = 72) fulfilling the same criteria were recruited along with the patients at the same period. The healthy controls included were from two categories, healthy family members from the patients’ households and unrelated individuals. Patients were randomly allocated to one of the two treatments: Synbiotic 2000 or placebo. Each participant was assessed at baseline (the day before treatment start) and post-treatment (within 2 weeks after the 9-week intervention was completed) through an interview and questionnaires on psychiatric and GI symptoms and non-fasting blood sampling between 8 am and 4 pm. All participants, research nurses, and data analysts were blind to the allocation until all analyses were completed. Out of 248 patients, 182 completed the 9-week intervention, and 156 patients provided blood samples at both baseline and follow-up. Controls were assessed at only one time-point, and 61 out of them provided blood samples. The study was approved by the Regional Ethical Review Board in Stockholm (2015/884-31/1 and 2017/91-31).

### 2.2. Interventions

Synbiotic 2000, the active treatment provided by Synbiotics AB Sweden for free, consisted of lyophilized 4 × 10^11^ CFU of three lactic acid bacteria, *Pediococcus pentosaceus 5-33:3/16:1* (Strain deposit number: LMG P20608), *Lactobacillus casei ssp paracasei F19* (LMG P-17806), *Lactobacillus plantarum 2362* (LMG P-20606), and 2.5 g of each of the fermentable fibers beta-glucan, inulin, pectin and resistant starch per dose. The composition has been shown to have anti-infectious and anti-inflammatory effects in several randomized controlled trials exemplified by [70,71,72], in particular, preventing gut leakage [73]. Placebo was maltodextrin which is an oligosaccharide without a prebiotic effect. All sachets were stored at −20 °C until 14 days before use. Patients were asked to follow the treatment with one dose per day for 9 weeks. No patient missed treatment for more than 20 days and never more than 4 days in a row [31].

### 2.3. Analysis of Plasma Immune Activity Markers

Peripheral blood was collected in tubes containing EDTA. Immediately after collection, the tubes were centrifuged at 1700× *g* (3500 rpm) for 20 min, and plasma was directly aliquoted into sterile cryotubes and stored at −80 °C until analysis. In total, 24 predesigned markers were measured via the Meso Scale Discovery (MSD, Meso Scale Diagnostics, Rockville, MD, USA) platform. The levels of CRP, serum amyloid A (SAA), sICAM-1, and sVCAM-1 were measured using VPLEX Vascular Injury Panel 2 Human Kit (Cat. #K15198D). Eotaxin-1, fractalkine, growth-regulated oncogene α (GRO-α), interferon (IFN)-γ, IL-1β, IL-2, sIL-2Rα, IL-6, IL-10, IL-12/IL-23p40, IL-17A, IL-16, IL-18, MCP-1, TNF-α, TNF-related apoptosis-inducing ligand (TRAIL) and vascular endothelial growth factor A (VEGF-A) were measured using U-PLEX Biomarker Group1 Human Multiplex Assays (Cat. #K15067L), and TGF-β1, TGF-β2, and TGF-β3 were measured using U-PLEX TGF-β Combo Human kits (#K15241K), according to the manufacturer’s instructions. In each plate, standard curves were generated using the manufacturer-provided calibrators in duplicates, and all the curves had a robust correlation (R^2^ > 0.999). Two inter-plate controls were kept in each plate: manufacturer-provided Vascular Injury Control 1 and 2 for VPLEX, and two self-designed samples were a pool of patient samples for the UPLEX assays. Each plasma sample was run in a single well, and five 96-well plates in total were run per analyte. Samples from the same ADHD patient, i.e., from both baseline and follow-up, were run in the same plate, and patient and control samples were distributed evenly across all plates. The lower limit of detection (LLOD) per analyte and plate was set to 2.5* the standard deviation of the background signal (Table S1). More than 25% of the detected values of IFN-γ, IL-1β, IL-2, IL-17A, and TNF-α were below LLOD; therefore, these five markers were excluded from the statistical analysis. Two analytes (IL-6 and IL-10) had a few data points with detected values below LLOD, and these values were replaced by the LLOD of the corresponding analyte and plate (Table S1). The plasma sample values obtained from the other analytes were all within the detection range. The median (range) of the within-plate coefficients of variation (CV) from the calibrators was 2.54% (1.10–5.28%), and the between-plate CV from inter-plate controls was 9.93% (4.63–16.9%) for the 19 analytes included in the statistical analysis (Table S1). All plasma samples had undergone two freeze/thaw cycles. To exclude major circadian rhythmicity of the analytes, we plotted their levels by day-time of sampling. No major change in level over time was detected for any analyte (Figure S1A), for sICAM-1 in agreement with Wipfler et al. [74].

### 2.4. Analysis of Plasma Short-Chain Fatty Acids (SCFAs)

SCFAs (formic, acetic, propionic, butyric, isobutyric, succinic, valeric, isovaleric, and caproic acid) were analyzed in EDTA plasma by liquid chromatography–mass spectrometry (LC-MS) according to a method described previously [75] with some modifications at Department of Biology and Biological Engineering, Chalmers University of Technology, Gothenburg (details in Supplementary Materials). Forty-two samples in singlets from both ADHD patients (baseline and follow-up from the same person) and controls were run in each batch. In total, eleven batches were analyzed in two rounds (6 batches in the first round in March 2020 and 5 batches in the second round in July 2020). Twenty-two patient samples were analyzed in both rounds, selected to cover the range of the values in the first round. Three SCFAs (isobutyric, valeric, and caproic acid) were excluded from data analysis because of the poor correlation detected by the twenty-two rerun samples, leaving six SCFAs for statistical analyses (Figure S1B). All plasma samples for the analysis had undergone two freeze/thaw cycles. For each batch, two quality controls (QCs) for each analyte with levels in the range found in our patient samples were run in triplicates and were used to calculate the within-batch CV being 9% (5–11%) for the six SCFAs. The between-batch variation for the two rounds was controlled by normalizing the sample values with the same QCs kept in each batch. The normalization ratio for each analyte per batch was calculated as (mean of the QC values of the individual batch)/(mean of the total QC values from all batches run in the same round). All statistical analyses for SCFAs were performed on normalized data. Because plasma levels of acetate, propionate, and butyrate were reported to peak approximately 7 h after colonic administration of SCFAs [76], we tested if there was any apparent peak in plasma levels of these SCFAs, which would appear plausibly at 1–4 pm as a consequence of breakfast. We could not detect any indication of the major influence of breakfast (Figure S1A).

### 2.5. Cell Culture

Human aortic vascular smooth muscle cell line (hAVSMCs), developed from a 23 years old African American healthy female, was purchased from (ATCC, Manassas, VA, USA, https://www.atcc.org). The cells were cultured in DMEM (cat. #12320032, Thermo Fisher Scientific, Waltham, MA, USA) supplemented with 10% FBS, 1% Na Pyruvate, 2.5% penicillin/streptomycin, 1% FungiZone, 1% L-glutamine and 1% HEPES (cat. #A3840002, Thermo Fisher Scientific). All the cells used for experiments were from passages 9–13. Confluent cells from each of the three independent experiments were split into 21 wells (12-well plates). After seeding, cells were cultured overnight and then incubated with PBS (control), formic acid (500 μM and 50 mM), acetic acid (50 μM and 5 mM), or propionic acid (50 μM and 500 μM) for 24 h, whereafter IL-1β (2.5 ng/mL) was added. After 8 h, the cells were harvested in Trizol Reagent (cat. #15596018, Invitrogen, Waltham, MA, USA) for investigating ICAM-1 expression determined using qRT-PCR (see Supplementary Materials).

### 2.6. Statistical Analysis

Analysis of differences in analyte levels between diagnosis groups (control versus ADHD patient), age groups (child versus adult), medication groups (yes versus no), and sexes (males versus females) was performed using Mann–Whitney U tests. Statistical relationships between two analytes were assessed by applying Spearman’s rank correlation tests. Descriptive statistics are presented with median levels and IQR. To correct for multiple testing, false discovery rate (FDR)-adjusted *p* values (q values according to the Benjamini–Hochberg method) are reported for immune activity markers, and statistical significance was set at αFDR = 0.05. As the SCFA concentrations formed only 3 non-correlated groups, FDR was not applied on SCFA data, but statistical significance was set at α = 0.017 (Bonferroni correction, 0.05/3 = 0.017) (Figure S2). The levels of analytes in plasma were generally not normally distributed and were, therefore, naturally logarithm (ln) transformed for use in the parametric statistical analysis. A Synbiotic 2000 intervention effect, compared to placebo, was assessed separately for children and adults, applying analysis of covariance (ANCOVA) on ln-transformed analyte levels at follow-up, adjusting for sex and baseline levels of the analyte. Treatment effect estimates with 95% and 99% confidence intervals (CIs) of analyte levels are reported. Sensitivity analyses of intervention effects on analyte levels were performed in subgroups stratified by ADHD medication or plasma level at baseline of sVCAM-1 (high versus low). Statistical significance in the intervention effect models was set at α = 0.01, while suggestive statistical significance was set at α = 0.05. A list of both 95% CIs and 99% CIs for intervention effects is shown in Table S2. All statistical analyses were performed using R programming language version 3.6.3 (Posit, Boston, MA, USA). Graphs were made using the ggplot2 package from R [77].

## 3. Results

### 3.1. Baseline Levels of Immune Activity Markers in ADHD Patients

**Figure 1:**
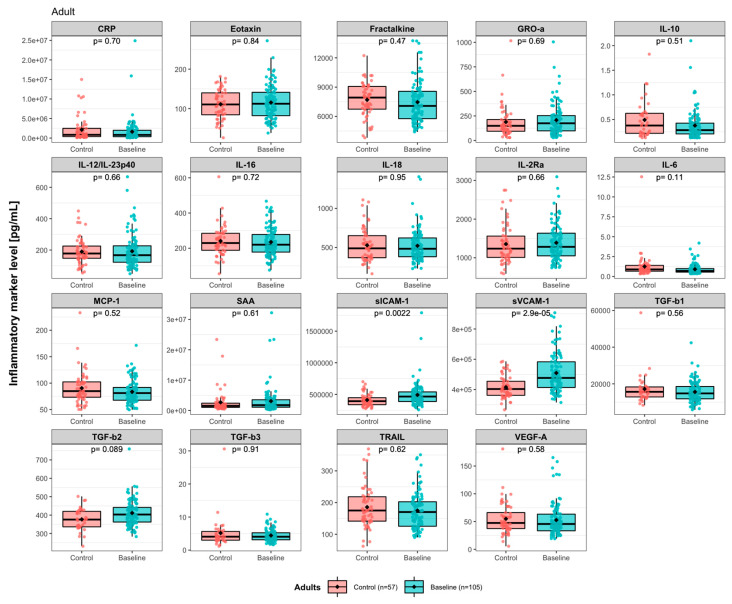
Immune activity marker level comparisons between adult healthy controls and adult ADHD patients: Y-axes represent analyte levels. The differences between groups were analyzed using nonparametric Mann–Whitney U test, and FDR-adjusted *p* values are shown. Each dot represents a participant. Outliers (three for CRP and two for SAA) were excluded by the defined cutoff of more than 50* interquartile range (IQR) from the median.

Clinical characteristics of the study participants, being pediatric and adult ADHD patients and adult healthy controls, are summarized in Table 1 and Table S3. We measured the levels of 24 predesigned immune activity markers in plasma from the patients before (baseline) and after (follow-up) intervention. Nineteen markers out of the 24 had detectable levels and were included in the data analysis (Table S1). High correlations were observed between many of the 19 markers, especially within the pairs CRP/SAA, sICAM-1/sVCAM-1, and GRO-α/TGF-β1 in both healthy controls and patients (Figure S3). In adults, the baseline levels of pro-inflammatory sICAM-1 (FDR-adjusted *p*[*pFDR*] = 0.0022) and sVCAM-1 (*pFDR* = 2.9 × 10^−5^) were significantly higher in ADHD patients compared to healthy control (Figure 1).

**Figure 2:**
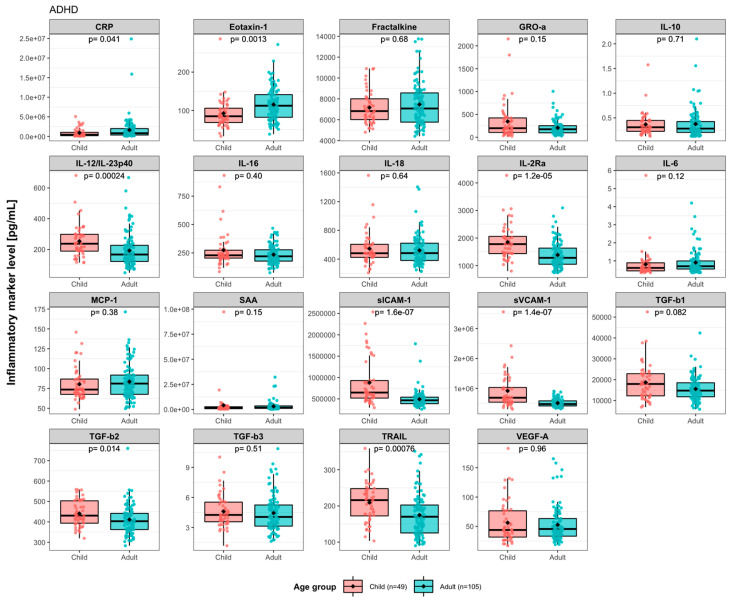
Differences in immune activity marker level between children and adults with ADHD at baseline. *Y*-axes represent analyte levels. The differences between groups were analyzed using nonparametric Mann–Whitney U test, and FDR-corrected *p* values are shown. Each dot represents a participant.

The healthy controls included were from two categories, healthy family members and unrelated individuals. The baseline levels of eotaxin-1, fractalkine, MCP-1, TGF-β3, and TRAIL differed between these two groups of controls (Figure S4A). When comparing ADHD to the two categories of controls separately, we found that the baseline levels of MCP-1 (*pFDR* = 0.026), fractalkine (*pFDR* = 0.050), TGF-β3 (*pFDR* = 0.049), and TRAIL (*pFDR* = 0.039) were lower in ADHD as compared to family members only, and eotaxin-1 (*pFDR* = 0.042) were higher as compared to unrelated controls only (Figure S4B). Due to a small sample size of children controls (n = 4, age: 12–14 years), we could not determine any case-control difference for children. Comparing baseline levels between children and adults with ADHD revealed significant differences for 8 of the 19 markers (Figure 2). Among them, IL-12/IL-23p40 (*pFDR* = 2.4 × 10^−4^), IL-2Rα (*pFDR* = 1.2 × 10^−5^), sICAM-1 (*pFDR* = 1.6 × 10^−7^), sVCAM-1 (pFDR = 1.4 × 10^−7^), TGF-β2 (*pFDR* = 0.014) and TRAIL (*pFDR* = 7.6 × 10^−4^) levels were higher in children, while CRP (*pFDR* = 0.041) and eotaxin-1 (*pFDR* = 0.0013) levels were higher in adults. This suggests that children and adults with ADHD have different profiles of immune activity marker levels in plasma. In addition, sex-disaggregated statistical analyses showed that the levels of IL-10 were higher in boys vs. girls with ADHD, while eotaxin-1 (*pFDR* = 0.0037) and IL-16 (*pFDR* = 0.024) were higher and IL-12/IL-23p40 (*pFDR* = 0.0037) and SAA (*pFDR* = 0.00037) were lower in adult males vs. females with ADHD (Figure S4C). Patients were randomly allocated into either of the two treatment groups, and we could, at baseline, not detect any difference in the markers mentioned between the two treatment groups for neither children nor adults (Figure S4D).

### 3.2. Effects of Synbiotic 2000 on Immune Activity Markers

**Figure 3:**
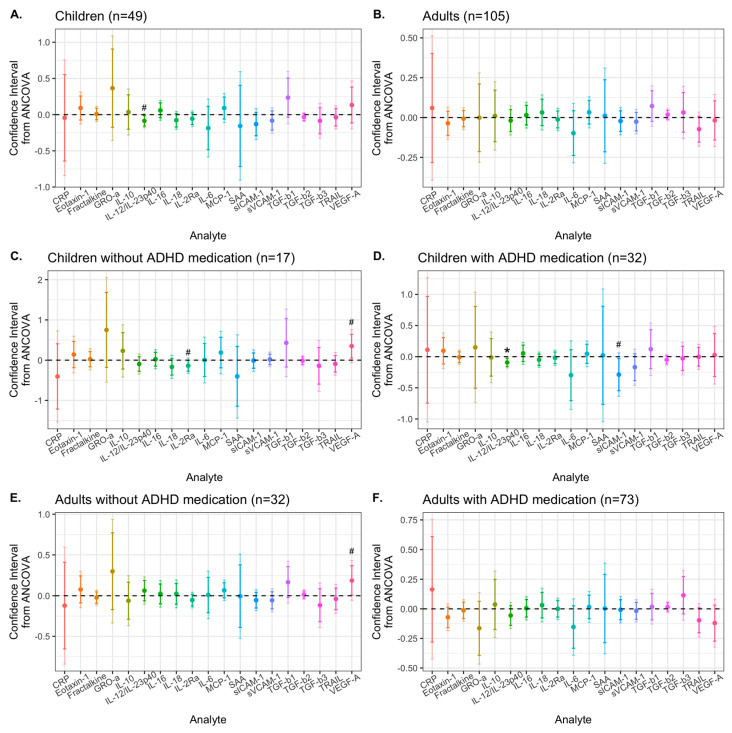
Confidence intervals (CIs) for treatment effects of Synbiotic 2000, compared to placebo and adjusted by sex, on immune activity marker levels. CIs were from analysis of covariance models for (**A**) children; (**B**) adults; (**C**) children on ADHD medication during the last 3 months; (**D**) children not on ADHD medication during the last 3 months; (**E**) adults on ADHD medication during the last 3 months; (**F**) adults not on ADHD medication during the last 3 months. Dark colors indicate 95% CIs, and light colors indicate 99% CIs. ADHD medication includes methylphenidate, dexamphetamine, atomoxetine, and for adults also lisdexamphetamine. A CI below 0 means that Synbiotic 2000, compared to placebo, reduced the analyte levels. * Statistical significance (α = 0.01); # difference at α = 0.05; Outliers (three for CRP and two for SAA) were excluded by the defined cutoff of more than 50* interquartile range (IQR) from the median.

Children treated with Synbiotic 2000 showed a significant reduction of levels of IL-12/IL23p40, sICAM-1, and TGF-β3 from baseline to 9-week follow-up, while children treated with placebo did not show any level change of any immune activity marker over time (Figure S5A and Table S4). Among adults, participants from both the placebo and Synbiotic 2000 groups had reduced sICAM-1 and sVCAM-1 levels at 9 weeks, while only the placebo group showed reduced TGF-β2 and TGF-β3 levels over time (Figure S5A). The treatment effect of Synbiotic 2000, compared to placebo, on analyte levels was analyzed with ANCOVA adjusted by sex and baseline levels of the analyte (Figure 3A–D).

Among children with ADHD, pro-inflammatory cytokine IL-12/IL-23p40 was reduced by Synbiotic 2000 compared to by placebo, at α = 0.05, defined as the suggestive difference (95% CI: −0.158, −0.014, *p* = 0.020) (Figure 3A). Since our previous study showed that current (or last three months) ADHD medication in children is associated with elevated levels of the vascular inflammatory markers sICAM-1 and sVCAM-1 [67] (Yang et al., 2020a) (Figure S4E, *pFDR*-child = 0.050), we stratified the analysis by current ADHD medication [yes/no]. For children who were currently on ADHD medication, Synbiotic 2000 manifested a significant reduction of IL-12/IL-23p40 (99% CI: −0.180, −0.005, *p* = 0.0070) and a suggestive reduction in sICAM-1 levels (95% CI: −0.547, −0.030, *p* = 0.030) compared to placebo (Figure 3D). For children not currently on ADHD medication, sIL-2Rα was suggestively reduced (95% CI: −0.274, −0.001, *p* = 0.049). For children and adults who were not currently on ADHD medication, Synbiotic 2000 suggestively increased the levels of VEGF-A (children: 95% CI: 0.054, 0.644, *p* = 0.024; and adults: 95% CI: 0.007, 0.368, *p* = 0.043) (Figure 3C,E and Figure S5B). This suggestive VEGF-A increase in children without ADHD medication was, however, probably driven by a VEGF-A reduction in the placebo group (*p* = 0.21, n = 7, Figure S5B). As the effect of Synbiotic 2000 in children on ADHD medication may be because of elevated sVCAM-1 levels at baseline, we explored the effect on child and adult patients with baseline sVCAM-1 levels above the median (cut off = 519,519.7 pg/mL). As expected, for children, the effects of Synbiotic 2000 vs. placebo in the high sVCAM-1 group were similar to those in the ADHD medication group (data not shown). In adults, however, those with high sVCAM-1 level had a suggestive reduction of sVCAM-1 (95% CI: −0.245, −0.007, *p* = 0.039) and sIL-2Rα (95%CI: −0.145, −0.017, *p* = 0.015) by Synbiotic 2000 compared to placebo (Figure S6A). Adults with low sVCAM-1 levels had a suggestive reduction of IL-6 (95% CI: −0.359, −0.011, *p* = 0.037) (Figure S6B), which was partially driven by the placebo effects (Figure S6C).

### 3.3. Baseline Levels of Short-Chain Fatty Acids (SCFAs) in ADHD Patients

**Figure 4:**
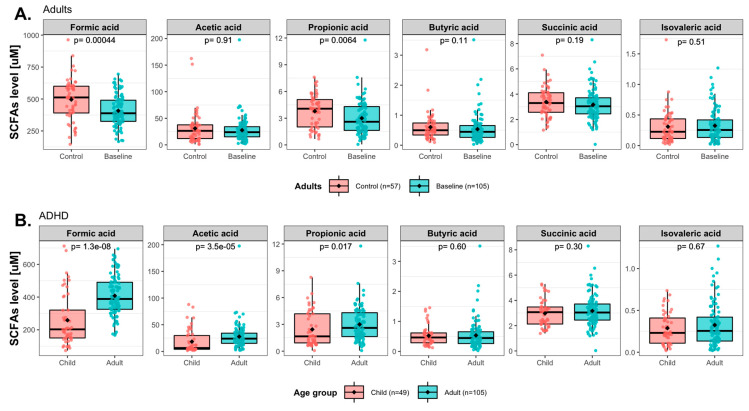
SCFA level comparisons at baseline (**A**) between healthy controls and ADHD patients for adults and (**B**) between children and adults with ADHD. *Y*-axes represent analyte levels. The differences between groups were analyzed using nonparametric Mann–Whitney U test and FDR-adjusted *p* values are shown. Each dot represents a participant.

Plasma concentrations of six SCFAs were analyzed in the ADHD patients and the healthy controls. We found that the shorter SCFAs (formic acid, acetic acid, propionic acid, and succinic acid) were significantly correlated with each other in both controls and patients (Figure S2). The statistical significance was set at α = 0.017, which was corrected for 3 independent tests of the 6 SCFAs. In adults, ADHD patients had significantly lower baseline concentrations of formic acid (*p* = 4.4 × 10^−4^) and propionic acid (*p* = 0.0064) as compared to healthy controls (Figure 4A). Furthermore, baseline acetic and propionic acids (*p* = 2.5 × 10^−4^, *p* = 0.0010 respectively) concentrations were significantly higher in family controls than in unrelated controls (Figure S7A). The baseline concentrations of formic (*p* = 0.00011), acetic (*p* = 0.016), propionic (*p* = 4.3 × 10^−5^), and butyric acids (*p* = 0.014) were all lower in adults with ADHD as compared to adult family controls, while acetic acid concentrations (*p* = 0.0043) were higher in ADHD as compared to adult unrelated controls (Figure S7B).

Comparing levels between pediatric and adult ADHD patients, concentrations of formic acid (*p* = 1.3 × 10^−8^), acetic acid (*p* = 3.5 × 10^−5^), and propionic acid (*p* = 0.017) were significantly lower in children than in adults (Figure 4B), which suggests that among those with ADHD children and adults have different SCFA profiles in plasma.

### 3.4. Effects of Synbiotic 2000 on SCFAs

**Figure 5:**
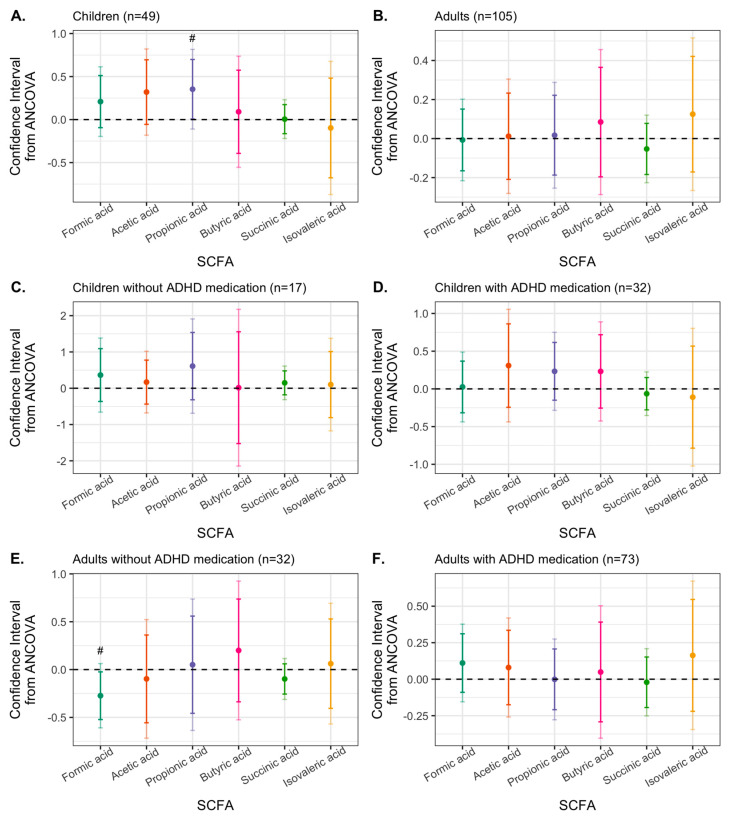
Confidence intervals (CIs) for treatment effects of Synbiotic 2000, compared to placebo and adjusted by sex, on SCFAs. CIs were from analysis of covariance models among (**A**) children; (**B**) adults; (**C**) children on ADHD medication during the last 3 months; (**D**) children not on ADHD medication during the last 3 months; (**E**) adults on ADHD medication during the last 3 months; (**F**) adults not on ADHD medication during the last 3 months. Dark colors indicate 95% CIs, and light colors indicate 99% CIs. ADHD medication includes methylphenidate, dexamphetamine, atomoxetine, and for adults also lisdexamphetamine. A CI below 0 means that Synbiotic 2000, compared to placebo, reduced the analyte levels. * Statistical significance (α = 0.01); # difference at α = 0.05.

No significant changes in SCFA concentrations from baseline to follow-up were found neither for those on placebo nor those treated with Synbiotic 2000 for neither children nor adults (Figure S8A). However, treatment effects on SCFA levels comparing the two interventions were analyzed using ANCOVA, applying similar models as those used for analyzing treatment effects on immune activity markers. We found that Synbiotic 2000, compared to the placebo, suggestively increased propionic acid concentrations in children with ADHD (95% CI: 0.006, 0.699, *p* = 0.046.) (Figure 5).

As shown in Figure S7E, the concentrations of propionic acid were lower among the children with current ADHD medication than those without ADHD medication (*p* = 0.0057). In the sensitivity analysis where we stratified for ADHD medication, we found that Synbiotic 2000, compared to placebo, suggestively reduced formic acid levels in adults who were not on ADHD medication at sampling (Figure 5 and Figure S8B).

### 3.5. Associations between Immune Activity Markers and SCFAs

**Figure 6:**
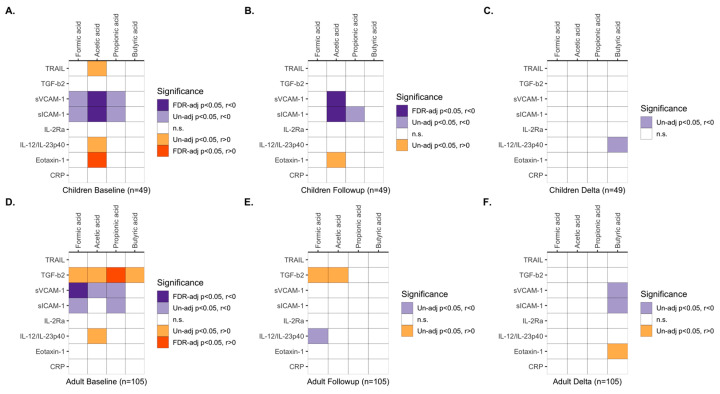
Correlation analysis between levels of immune activity markers and SCFAs in ADHD. Spearman’s rank correlation coefficients for (**A**) children before treatment; (**B**) children after treatment; (**C**) children for the change (follow up minus baseline) during the treatment; (**D**) adults before treatment; (**E**) adults after treatment; (**F**) adults for the change (follow up minus baseline) during the treatment. FDR-adjusted significant correlations (*p* < 0.050) are indicated in dark colors, and unadjusted correlations (*p* < 0.050) are indicated in light colors. Red indicates a positive correlation, and blue indicates negative correlation.

Immune activity markers and SCFAs are both important components in the microbiota–gut–brain axis, and a small number of cellular in vitro studies have reported the effects of butyrate on a few immune activity markers. We performed a correlation analysis between plasma levels of the immune activity markers and concentrations of the SCFAs, which we, in the aforementioned analyses, found to be different in ADHD compared to controls (all controls for immune activity markers and family controls for SCFAs). In children with ADHD, baseline acetic acid levels were significantly negatively correlated with pro-inflammatory sICAM-1 and sVCAM-1 (*pFDR* < 0.050), while formic and propionic acid levels were suggestively negatively correlated with sICAM-1 and sVCAM-1 (*p* < 0.050). Further, baseline acetic acid in children correlated positively with pro-inflammatory eotaxin-1 (*pFDR* < 0.050) and suggestively positively to IL-12/IL-23p40 and TRAIL (*p* < 0.050) (Figure 6A and Figure S9); however, the eotaxin-1 levels were lower than those seen in adult patients and controls (Figure 1). The significant negative correlation for acetic acid with sICAM-1 and sVCAM-1, and suggestive negative correlation between propionic acid and sICAM-1, in children, were also detected at follow-up (Figure 6B and Figure S9). The negative correlations of the SCFAs with sVCAM-1 and sICAM-1 were in part indicated also in adult patients at baseline (Figure 6D). Additionally, TGF- β2 levels correlated positively with propionic acid concentrations in adult patients but were within the range of the levels in the controls (Figure 1).

### 3.6. Effects of SCFAs on ICAM-1 Expression in Human Aortic Vascular Smooth Muscle Cells In Vitro

To further validate the negative correlations between SCFAs and sICAM-1, we did three independent in vitro experiments in human aortic vascular smooth muscle cells. Our results showed lower IL-1β-induced ICAM-1 expression when the cells were pre-incubated with formic acid, acetic acid, or propionic acid of the concentrations found in plasma (Figure S10).

## 4. Discussion

**Figure 7:**
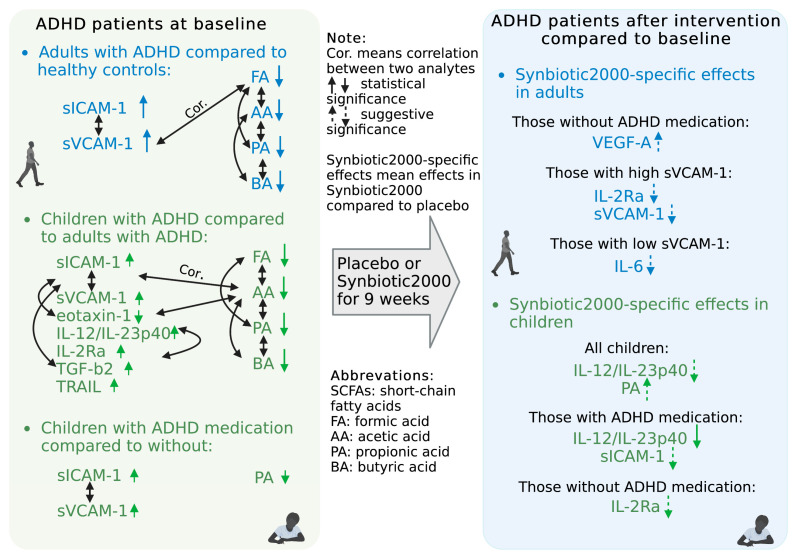
Summary of the findings.

This study is the first to report the effects of a synbiotic intervention on plasma levels of immune activity markers and SCFAs in children and adults with ADHD. We previously reported that this intervention in an RCT design reduced autistic traits in children and improved emotion regulation in adults with ADHD [31]. Now, we report that there was no statistically significant overall effect of Synbiotic 2000 compared to placebo on any analyte analyzing all the pediatric and all adult participants as one group. However, age-group-stratified analyses are more appropriate as plasma levels of several of the analytes were at baseline different in the children compared to in the adults. Actually, in children the Synbiotic 2000 intervention, compared to the placebo, suggestively reduced pro-inflammatory IL-12/IL-23p40 levels. As children on ADHD medication have previously been reported to have higher levels of the pro-inflammatory adhesion molecules sICAM-1 and sVCAM-1 than children without ADHD medication and adults with ADHD [67], we analyzed this pediatric group on ADHD medication separately. In children on ADHD medication Synbiotic 2000, compared to placebo, reduced IL-12/IL-23p40 levels significantly and reduced sICAM-1 levels suggestively. In children without ADHD medication, Synbiotic 2000, compared to placebo, suggestively reduced IL-2Rα levels. We cannot determine if the children’s IL-12/IL-23p40, sICAM-1, or IL-2Rα levels at baseline were higher than that of healthy controls in this age group, although the controls’ levels were low (Figure S5A), as n_controls_ is only 4. However, we show that children with ADHD at baseline have higher IL-12/IL-23p40 and IL-2Rα levels than adults with ADHD, and children on ADHD medication have higher sICAM-1 levels than ADHD children without medication and adults. A previous report has shown childhood IL-12/IL-23p40 levels to be lower than adulthood levels [78]. This suggests that children with ADHD do have abnormally high IL-12/IL-23p40 levels. However, we cannot exclude the possibility that higher baseline levels in the children of sICAM-1 [79] and IL-2Rα are normal. To explore a potential link for Synbiotic 2000 to IL-12/IL-23p40, sICAM-1, and IL-2Rα levels in pediatric ADHD patients, we assessed plasma levels of the bacterial fermentation metabolites SCFAs. Synbiotic 2000, compared to placebo, suggestively elevated plasma levels of propionic acid in the children, and the correlations between the shortest SCFAs: formic acid, acetic acid, and propionic acid, were very strong. Moreover, the levels of formic, acetic, and propionic acid correlated negatively with levels of sVCAM-1 and/or sICAM-1, and the latter two correlated strongly with each other. The levels of formic, acetic, and propionic acid in children were at baseline lower than in adults with and without ADHD (Figure 4A,B), and at least propionic acid levels appeared low compared to healthy control children (Figure S8A). Altogether, this proposes that elevating the highly correlated formic, acetic acid, and propionic acid might alleviate an sICAM-1-marked vascular inflammation in children with ADHD (Summarized in Figure 7). In support, our preliminary results from in vitro experiments with human aortic vascular smooth muscle cells showed that pre-incubation with formic, acetic, or propionic acid tended to reduce the expression of ICAM-1 induced by IL-1β (Figure S10). However, there was at baseline a suggestively significant positive correlation between levels of propionic acid and IL-12/IL-23p40 (Figure 6), indicating that the SCFAs did not mediate the Synbiotic 2000-induced reduction of IL-12/IL-23p40 levels.

IL-12 and IL-23 are heterodimers and share the p40 subunit called IL-12/IL-23p40. IL-12 and IL-23 promote Th1 and Th17 expansion, respectively, and are reported to be involved in the pathology of inflammatory bowel disease (IBD). The p40 subunit is a therapeutic target in IBD [80,81]. GI symptoms are overrepresented in ADHD [82], which is also the case in our cohort [67]. The adhesion molecules ICAM-1 and VCAM-1 are expressed predominantly by endothelial cells. ICAM-1 participates in binding leukocytes to the endothelial cell, and VCAM-1 participates in the subsequent leukocyte extravasation into the surrounding tissue. sICAM-1 and sVCAM-1 are the soluble isoforms of ICAM-1 and VCAM-1, respectively, found at plasma levels in proportion to endothelial cell membrane-bound levels [83]. They have key roles in regulating the immune homeostasis in the gut endothelium; both sICAM-1 and sVCAM-1 have been reported upregulated in IBD patients [84,85], and sICAM-1 levels were found to reduce the mucosal healing process in patients with Crohn’s disease [86]. Higher levels of ICAM-1 and VCAM-1 have also been associated with schizophrenia, depression, and bipolar disorder, and interestingly, higher ICAM-1 levels have been associated with BBB hyper-permeability [58,87]. IL-2Rα is like the other IL-2R subunits expressed by Treg cells and recently activated T cells, and elevated plasma levels of soluble IL-2Rα indicate ongoing pro-inflammatory immune activity and are reported in mood disorders, schizophrenia, and ASD [48,49,50]. Several RCTs of synbiotics or probiotics have previously been reported to reduce endothelial adhesion molecules and IL-12/IL23p40 in cardiometabolic disorders and IBD [88,89,90,91,92]. Moreover, an RCT conducted in patients with ulcerative colitis showed that butyrate enemas significantly increased the colonic IL-10/IL-12 ratio in mucosal biopsies, however not significantly when compared to the placebo group [93]. Butyrate was reported to suppress IL-12p40 mRNA accumulation and massively enhance IL-10 secretion in primary human monocytes [94]. Moreover, both butyrate and propionate were reported to inhibit the ICAM-1 and VCAM-1 expression in human endothelial cells in vitro [95,96]. However, the SCFA levels used in these models were higher than the physiological levels in human body fluids. Our preliminary results from cell culture experiments with human aortic vascular smooth muscle cells show supportive results of the anti-inflammatory potential of these SCFAs. Pre-incubation with formic, acetic, or propionic acid at the concentrations detected in human plasma tended to prevent the IL-1β-induced ICAM-1 expression (Figure S10). However, we did not analyze the cellular effects of butyrate, nor the effects on IL-12/IL-23 or IL-2Rα, in this study. IL-12 was previously shown to enhance IL-18-induced ICAM-1 expression in human monocytes [97]. Further studies using cell culture bioassays are needed to understand the complexity between physiological levels of SCFAs and inflammatory response.

Notably, the pattern of correlations between levels of immune activity markers and SCFAs detected in ADHD patients was not found in controls, suggesting that the associations between immune activity analytes and SCFAs are not generalizable beyond ADHD but depend on a complex regulation at physiological conditions (Figure 6 and Figure S11). However, in adults, there was no statistically significant or suggestive Synbiotic 2000 treatment effect in the whole group. Here, not only those treated with Synbiotic 2000 intervention but also those on placebo had a reduction of sICAM-1 and sVCAM-1 levels from baseline to follow-up (Figure S5A). Dietary change could not explain this placebo effect as there was dietary change for only beta-carotene between baseline and follow-up among the 57 nutrients [31]. However, 72.3% of the adult patients and 42.9% of the child patients were on dietary supplements, such as vitamins, omega-3, and probiotics, already at baseline and kept throughout the study (Table S3). Children on ADHD medication had higher sICAM-1 and sVCAM-1 levels [98]. An anti-inflammatory effect by Synbiotic 2000 may be more detectable when having limited group variation of baseline inflammatory state. That may partly explain why more suggestive effects of Synbiotic 2000 were seen in the children when stratifying for ADHD medication use. Accordingly, in adult patients with baseline sVCAM-1 levels above the median, Synbiotic 2000, compared to placebo, suggestively reduced IL-2Rα and sVCAM-1 levels. The detected reduction of IL-6 levels in those with baseline sVCAM-1 below the median may be explained by increases in the placebo group (Figure S6C). Thus, like in children with ADHD, our data suggest an effect of Synbiotic 2000 reducing certain markers involved in vascular inflammation in adult ADHD patients with elevated sVCAM-1 at baseline. In addition, Synbiotic 2000 suggestively reduced formic acid in the adults, not on ADHD medication; however, it was not supported by any effect on levels of immune marker that formic acid levels correlated with.

To our knowledge, we are the first to report plasma levels of fractalkine, GRO-α, IL-12/IL-23p40, IL-18, IL-2Rα, TGF-β1, TGF-β2, TGF-β3, TRAIL, and VEFG-A in individuals with ADHD. The adult ADHD patients displayed at baseline different levels only of pro-inflammatory sICAM-1 and sVCAM-1 compared to the whole control group. Compared to the adults with ADHD, the children with ADHD had different baseline levels of eight of the immune activity markers and were hence analyzed separately. Most of these differences in marker levels between children and adults are not previously reported, neither in ADHD patients nor healthy individuals. Levels at baseline of eotaxin-1, fractalkine, MCP-1, TGF-β3, and TRAIL were higher in healthy family members of the ADHD patients than among healthy unrelated controls. Both genetic and environmental underpinnings may explain this, although there is no report showing that these markers are higher in persons with ADHD. The plasma SCFA concentrations at baseline of the patients and family controls of this study were previously reported to show lower levels of plasma formic and propionic acid in adults with ADHD compared to family controls after controlling for antibiotic drug exposure and other potential influencing factors [98]. Accordingly, we now report that the baseline levels of these same SCFAs (formic and propionic acids) are in adults with ADHD lower compared to the whole control group but at similar levels as unrelated controls (Figure 4). Most studies on SCFAs in other neuropsychiatric disorders have analyzed SCFA levels in feces, which correlate poorly to SCFA levels in plasma, probably due to the significant uptake of certain SCFAs in the intestine [42]. A review on fecal SCFAs in children with autism showed poorly consistent findings between studies [99]. Additionally, most studies on SCFAs have focused on acetic, propionic, and butyric acid only [34,100]. An altered SCFA profile would indicate different dietary habits and/or different bacterial gut microbiomes, being established for autism [101] and proposed for ADHD [16].

The sample size of our RCT was relatively large, including 182 children and adults. An additional main strength is that the conducted in vitro experiments of SCFAs possible anti-inflammatory effect was at physiological SCFA levels. There are limitations to this study. First, there were only four healthy controls for children, and hence, we could not analyze this group. Therefore, we were unable to adequately relate the analyte levels in children with ADHD to reference values. Second, our data on medications with anti-inflammatory effects (melatonin, antidepressants, antipsychotics, anxiolytics, sleeping pills, proton-pump inhibitors, and statins) or medications with gut microbiome effects (melatonin, antipsychotics, or antidepressants) (Table S3) are not complete, as we lack information on the specific drug names and drugs specifically targeting inflammation; however, for the medication data that we do have, no associations with levels of the analytes were detected. Third, diet or diet supplements use during the intervention time could conceal the effects of Synbiotic 2000, although participants were asked not to change their diet from 4 weeks before baseline to follow-up, and we detected no relevant change over time in nutrients intake through a retrospective diet questionnaire [31]. Fourth, in the treatment effect analyses, we controlled only for sex.

## 5. Conclusions

This exploratory study revealed that persons with ADHD, especially children on ADHD medication, have higher-than-normal pro-inflammatory sICAM-1 and sVCAM-1 and lower SCFA levels in plasma and that children with ADHD also have higher levels of additional pro-inflammatory markers, e.g., IL-12/IL-23p40 and IL-2Rα. Treatment with Synbiotic 2000, compared to placebo, reduced IL-12/IL-23p40 levels and suggestively reduced sICAM-1 and IL-2Rα levels in children. Synbiotic 2000 also suggestively increased propionic acid levels, which, together with highly associated formic and acetic acid levels, in turn, correlated negatively with sICAM-1 and sVCAM-1 in the children and protected against IL-1β-induced sICAM-1 expression in vitro. This suggests that Synbiotic 2000, in children with ADHD, reduces markers of intestinal and vascular inflammation, the latter in part through increasing SCFA levels. The findings warrant further studies to determine if persons with ADHD would benefit inflammation-wise from dietary intake of Synbiotic 2000 or a similar synbiotic.

## Figures and Tables

**Table 1:** Characteristics at baseline of participants with ADHD.

		Children (n = 49)		Adults (n = 105)	
		Placebo (n = 21)	Synbiotic 2000 (n = 28)	Placebo (n = 54)	Synbiotic 2000 (n = 51)
		Median (IQR)/ N (%)	Median (IQR)/ N (%)	Median (IQR)/ N (%)	Median (IQR)/ N (%)
**Age [years]**		13 (8–18)	14 (8–18)	36 (19–51)	37 (19–55)
**Body mass index [kg/m^2^]**				22.8 (17.0–38.3)	24.3 (19.3–40.4)
**Sex**	Female	4 (19)	9 (32)	38 (70)	36 (71)
	Male	17 (81)	19 (68)	16 (30)	15 (29)
**Delivery route**	Vaginal	18 (86)	21 (75)	48 (89)	43 (84)
	C-section	3 (14)	4 (14)	5 (9)	7 (14)
	Acute C-section	0 (0)	1 (4)	1 (2)	1 (2)
	Unknown	0 (0)	2 (7)	0 (0)	0 (0)
**Breastfed**	<3 months	2 (10)	5 (18)	7 (13)	9 (18)
	3–6 months	0 (0)	4 (14)	12 (22)	10 (20)
	>6 months	18 (86)	17 (61)	29 (54)	22 (43)
	None	0 (0)	0 (0)	1 (2)	1 (2)
	Unknown	1 (5)	2 (7)	5 (9)	9 (18)
**Antibiotic drugs ^a^**	>3 times	0 (0)	1 (4)	3 (6)	1 (2)
	1–3 times	5 (24)	8 (29)	15 (28)	11 (22)
	None	16 (76)	18 (64)	34 (63)	36 (71)
	Unknown	0 (0)	1 (4)	2 (4)	3 (6)
**Melatonin medication**	Yes **†**	14 (67)	9 (32)	12 (22)	37 (73)
	No **†**	7 (33)	19 (68)	42 (78)	14 (27)
**ADHD medication ^b^**	Yes **†**	14 (67)	18 (64)	36 (67)	37 (73)
	No **†**	7 (33)	10 (36)	18 (33)	14 (27)
**Other prescribed drugs for adults ^c^**	Yes **†**	0 (0)	0 (0)	24 (44)	30 (59)
	No **†**	21 (100)	28 (100)	30 (56)	21 (41)
**Dietary supplements ^d^**	Yes	13 (62)	15 (54)	39 (72)	37 (73)
	No	8 (38)	13 (46)	15 (28)	14 (26)
**ICD-10 code ^e^**	F90.0	6 (29)	4 (14)	9 (17)	10 (20)
	F90.0B	12 (57)	18 (64)	28 (52)	31 (61)
	F90.0C	2 (10)	5 (18)	13 (24)	7 (14)
	F90.0X	0 (0)	0 (0)	2 (4)	2 (4)
	F90.1	1 (5)	0 (0)	0 (0)	0 (0)
	F90.8	0 (0)	0 (0)	1 (2)	0 (0)
	F98.8	0 (0)	1 (4)	1 (2)	1 (2)

Two persons were excluded from the study because of suspected acute infections based on high CRP (above 15 mg/mL) or SAA (above 10 mg/L) levels. No other person analyzed in this study had an obvious acute inflammation (their CRP levels were <15 mg/L). Results are given as median (25^th^–75^th^ percentile (IQR)) or as number (%) of subjects; **a**. number of antibiotic drug uses in the last two years (no one was on antibiotic drug use last 6 weeks); **b**. ADHD medications for children include the stimulants Methylphenidate (n = 14), Lisdexamphetamine (n = 10), the nonstimulant Atomoxetine (noradrenalin re-uptake inhibitor (n = 4)), and Methylphenidate plus Atomoxetine (n = 3), and for adults they include Methylphenidate (n = 34), Lisdexamphetamine (n = 34), Dexamphetamine (n = 12), Atomoxetine (n = 3) and Methylphenidate plus Atomoxetine (n = 1); **c**. other prescribed drugs for adults reported to influence immune activity and gut microbiome include antidepressants, antipsychotics, anxiolytics, sleeping pills (mainly antihistamines), proton-pump inhibitors and statins; **d**. supplements (e.g., vitamins, omega-3, probiotics) taken in the last 4 weeks. The probiotics used were *L. plantarum 299v* (1 child, 11 adults), Synbiotic 15 (similar constituents as Synbiotic 2000 but 15 × 10^9^ CFU instead of the 4 × 10^11^ CFU in Synbiotic 2000, 2 adults) and other (6 adults); **e**. 10^th^ revision of the International Statistical Classification of Diseases and Related Health Problems. **†** participants on medication currently or in last 3 months as “Yes”, not on medication currently or in the last 3 months as “No”.

## Data Availability

Restrictions apply to the availability of these data. Data are available from the corresponding author with the permission of the Swedish Ethical Review Authority.

## Supplementary Materials

The supporting information can be downloaded at: https://www.mdpi.com/article/10.3390/nu15051293/s1.
