# Supplementary material for: Effects of a Synbiotic on Plasma Immune Activity Markers and Short-Chain Fatty Acids in Children and Adults with ADHD—A Randomized Controlled Trial
Source: Nutrients. 2023 Mar 6;15(5):1293. doi: 10.3390/nu15051293 (PMC10004766; doi:10.3390/nu15051293)
Supplement: Supplementary file 1 [file nutrients-15-01293-s001.zip › nutrients-2204951-supplementary.pdf]

Supplementary information

Effects of a synbiotic on plasma immune activity markers and short-chain fatty acids in children and adults with ADHD —a randomized controlled trial

Liu L. Yang, MD PhD, Miranda Stiernborg, MSc, Elin Skott, Jingjing Xu, Yujiao Wu, PhD, Rikard Landberg, PhD, Samsul Arefin, Karolina Kublickiene, MD PhD, Vincent Millischer, MD PhD, Ida AK Nilsson, PhD， Martin Schalling, MD PhD, MaiBritt Giacobini, MD PhD, Catharina Lavebratt, PhD

Supplementary Methods

Analysis of plasma concentrations of short-chain fatty acids (SCFAs)
Analysis of ICAM-1 expression in human aortic vascular smooth muscle cells

Supplementary Tables and Figures

Supplementary **Figure S1.** (A) The relationship between sampling time and the levels of analytes; (B) Correlation analysis of SCFA levels between round 1 and round 2.
Supplementary **Figure S2**. Correlation matrix between six SCFAs.
Supplementary **Figure S3**. Correlation matrix between immune activity markers.
Supplementary **Figure S4**. Immune activity marker levels <u>at baseline</u> with respect to control type, sex, treatment group and ADHD medication stratified by age group.
Supplementary **Figure S5**. Plasma levels of immune activity markers: (A) Time effect (from baseline to the 9-week follow-up) for each intervention group (placebo and Synbiotic 2000); (B) Treatment effect (Synbiotic 2000 in comparison with placebo) stratified by ADHD medication.
Supplementary **Figure S6**. Plasma levels of immune activity markers: Treatment effect (Synbiotic 2000 compared to placebo) stratified by baseline plasma sVCAM-1 levels on in adults with ADHD.
Supplementary **Figure S7**. Short-chain fatty acid (SCFA) levels in plasma <u>at baseline</u> with respect to control type, sex, treatment group and ADHD medication stratified by age group.
Supplementary **Figure S8**. Plasma levels of short-chain fatty acid (SCFAs): (A) Time effect (from baseline to follow-up) on all SCFAs for each intervention group (placebo and Synbiotic 2000); (B) Treatment effect (Synbiotic 2000 in comparison with placebo) on formic acid in adults stratified by ADHD medication.
Supplemental **Figure S9**. Individual scatter plots of the correlations between plasma levels of immune activity markers and short-chain fatty acids (SCFAs) shown in Fig. 6 with un-adjusted $p < 0.05$.
Supplementary **Figure S10**. IL-1β-induced ICAM-1 mRNA expression in human aortic vascular smooth-muscle-cells after *in vitro* pre-incubation with different short-chain fatty acids (SCFAs).
Supplementary **Figure S11.** Correlation analysis between plasma levels of immune activity markers and short-chain fatty acids (SCFAs) in adult controls.

Supplementary Methods:

Analysis of plasma concentrations of short-chain fatty acids (SCFAs)

All reference compounds as well as analytical reagent-grade 3-nitrophenylhydrazine (3NPH)HCl (97%) and N-(3-dimethylaminopropyl)-N0-ethylcarbodiimide (EDC), HPLC grade pyridine, LC–MS grade acetonitrile, hypergrade LC-MS water and MeOH (Lichrosol) were purchased from Sigma–Aldrich. All reagents and solvents were used for a maximum of 5 days. ^{13}C6-3NPH was custom synthesized by IsoSciences Inc. (King of Prussia, PA, USA) and used as internal standard for all SCFAs. Plasma (10 µl) was incubated with 75% methanol (60 µl) and mixed with 200 mM 3-NPH (60 µl) and 120 mM EDC-6% pyridine (10 µl) at ambient temperature for 45 min under gentle shaking. The reaction was quenched by addition of 200 mM quinic acid (10 µl) at gentle shaking at ambient temperature for 15 min. The samples were centrifuged at 15 000 g for 5 min and the supernatant was moved to a new tube. The sample was made up to 1 mL by 10% methanol in water and again centrifuged at 15 000 g for 5 min. In total 100 µl of the derivatized (^{12}C) sample was mixed with 100 µl of labelled (^{13}C) internal standard. Samples were analyzed by a 6500+ QTRAP triple-quadrupole mass spectrometer (AB Sciex, 11432 Stockholm, Sweden) which was equipped with an APCI source and operated in the negative-ion mode. Chromatographic separations were performed on a Phenomenex Kinetix Core-Shell C18 (2.1, 100 mm, 1.7 um 100Å) UPLC column with SecurityGuard ULTRA Cartridges (C18 2.1mm ID) (changed at regular intervals). The column was backflushed for 60 min between each batch to ensure good chromatographic separation. LC-MS grade water (100% solvent A) and acetonitrile (100% solvent B) was the mobile phases for gradient elution. The column flow rate was 0.4 mL/min and the column temperature was 40°C, the autosampler was kept at 5°C. LC starting conditions at 0.5% B, held for 3 min, 3 min 2.5% B ramping linearly to 17% B at 6 min, then to 45% B at 10 min and 55% B at 13 min. Followed by a flush (100% B) and recondition (0.5% B), total runtime 15 min. The multiple reaction monitoring (MRM) transitions were optimized for the analytes one by one by direct infusion of the derivatives containing 50 mM of each fatty acid. The Q1/Q3 pairs were used in the MRM scan mode to optimize the collision energies for each analyte, and the two most sensitive pairs per analyte were used for the subsequent analyses. The retention time window for the scheduled MRM was 1 min for each analyte. The two MRM transitions per analyte, the Q1/Q3 pair that showed the higher sensitivity was selected as the MRM transition for quantitation. The other transition acted as a qualifier for the purpose of verification of the identity of the compound.

Analysis of ICAM-1 expression in human aortic vascular smooth muscle cells

Total cellular RNA was extracted using Direct-zol™ RNA MiniPrep Plus Kit (cat. #R2072, Zymo Research) according to the standard manufacturer's instructions. RNA concentration and purity (A260/A280) were measured using NanoDrop™ 2000 spectrophotometer (cat. #ND-2000). cDNA was synthesized directly after RNA extraction using SuperScript III First-Strand Synthesis System for qRT-PCR (cat. #18080051, Invitrogen) according to the standard protocol for cDNA synthesis with Veriti™ 96-Well Thermal Cycler (cat. #4375786, Applied Biosystems). qRT-PCR was carried out in a QuantStudio™ 6 Flex Real-Time PCR system (cat. #ZG21SCQSTUDIO6FLEX,

Applied Biosystems) using SYBR Green kit (cat. #4368702, Applied Biosystems) following the standard settings (5 µl SYBR green, 1 µl 10 µM primer pair, 4 µl cDNA). The specific primer pair sequences were for ICAM-1 (F: TGATGGGCAGTCAACAGCTA; R: GAAATTGGCTCCATGGTGAT) and glyceraldehyde-3-phosphate dehydrogenase (GAPDH) (F: CCTGCACCACCAACTGCTTA; R: CCAGTGAGCTTCCCGTTCAG), purchased from Invitrogen. The cycle threshold values for GAPDH were 18-20, and for ICAM-1 they were 22-30. All reactions were run in triplicates and normalized to reference gene GAPDH. Levels of ICAM-1 mRNA relative to that of GAPDH were calculated applying the $2^{-\Delta\Delta Ct}$ method using the average Ct value of the triplicates and the corresponding control triplicates as the calibrator with QuantStudio Software for 6 Flex.

Supplementary Figures and Tables:

A.

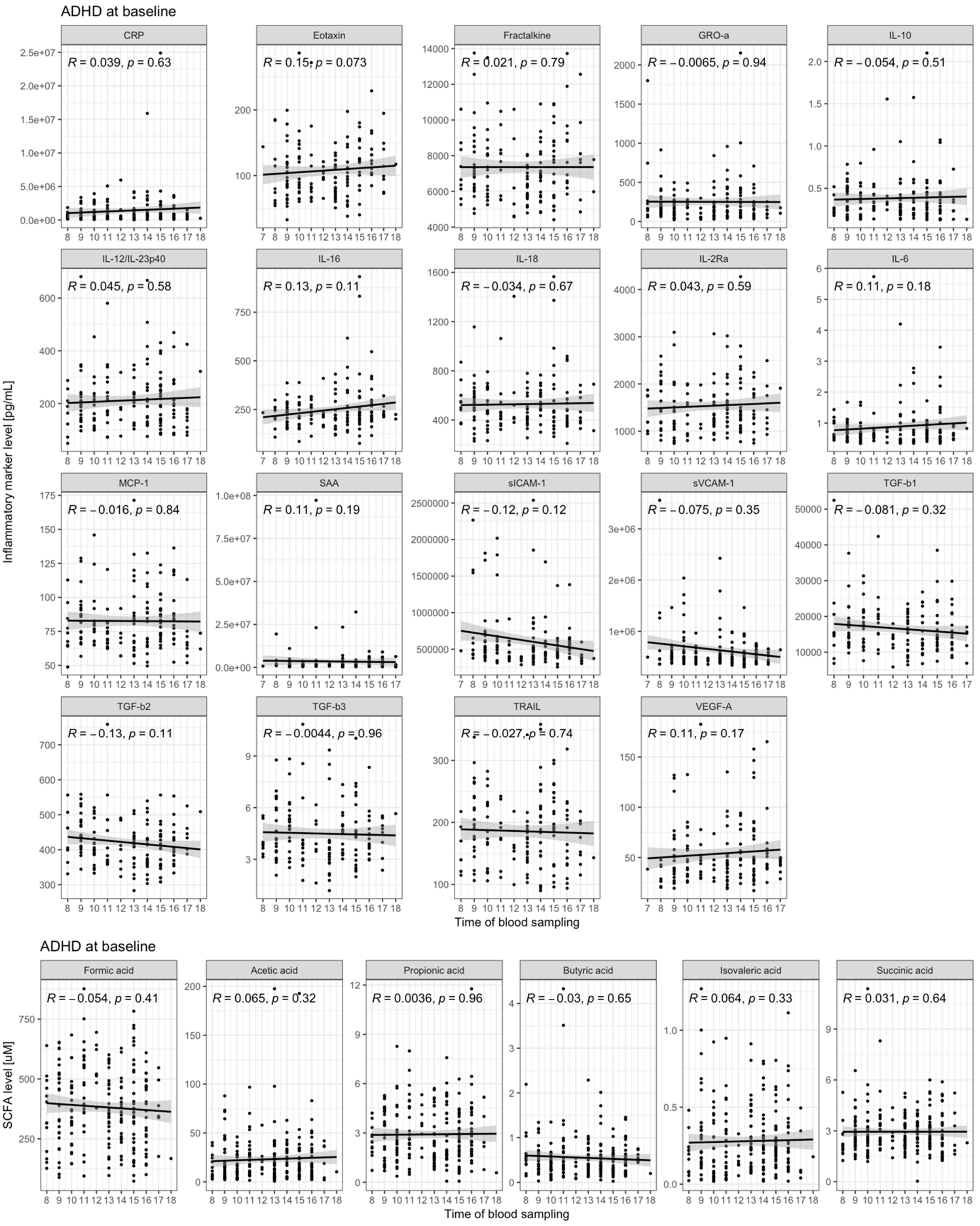

B.

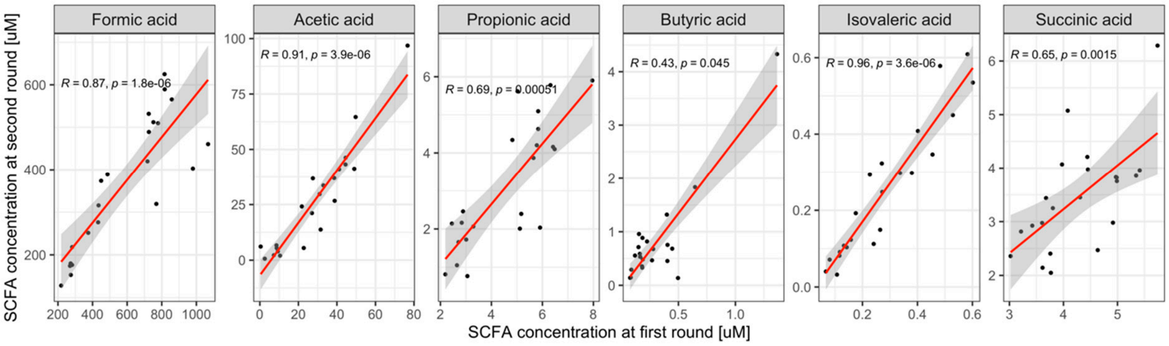

Figure S1. (A) The relationship between sampling time and the levels of analytes in ADHD patients at baseline; (B) Correlation analysis of SCFA levels between round 1 and round 2 from the samples (n=22) analyzed in two rounds. Spearman's rank correlation analysis. *r* and un-adjusted *p* values are shown.

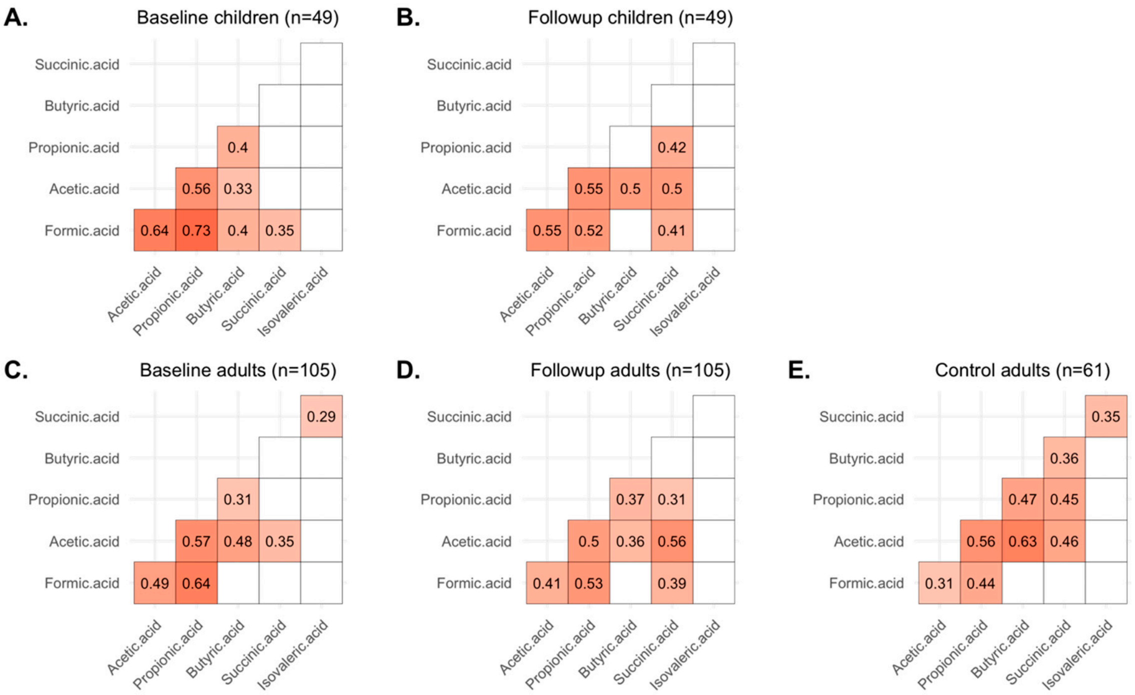

Figure S2. Correlation matrix between six SCFAs. Spearman's rank correlation coefficients for (**A**) child ADHD patients before treatment, (**B**) child ADHD patients after treatment, (**C**) adult ADHD patients before treatment, (**D**) adult ADHD patients after treatment, (**E**) adult healthy controls. FDR-adjusted significant correlations ($p < 0.050$) are shown with r values in the corresponding each box; Non-significant correlations are indicated by blank.

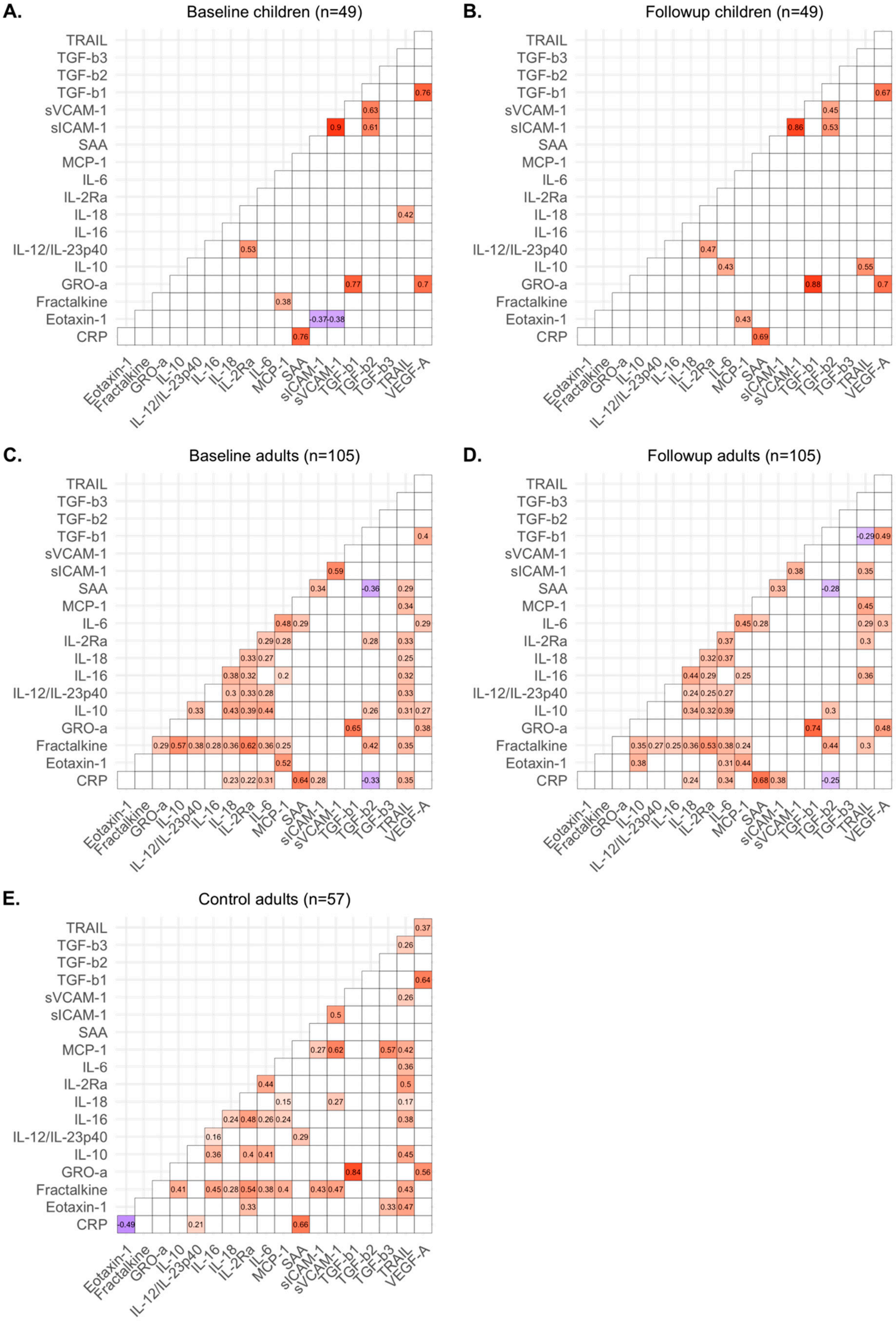
A.
Baseline children (n=49)
B.
Followup children (n=49)
C.
Baseline adults (n=105)
D.
Followup adults (n=105)
E.
Control adults (n=57)
TRAIL
TGF-b3
TGF-b2
TGF-b1
sVCAM-1
sICAM-1
SAA
MCP-1
IL-6
IL-2Ra
IL-18
IL-16
IL-12/IL-23p40
IL-10
GRO-a
Fractalkine
Eotaxin-1
CRP
VEGF-A

Figure S3. Correlation matrix between immune activity markers. Spearman's rank correlation coefficients for (**A**) child ADHD patients before treatment (**B**) child ADHD patients after treatment, (**C**) adult ADHD patients before treatment (**D**) adult ADHD patients after treatment, (**E**) adult healthy controls. FDR-adjusted significant correlations ($p < 0.050$) with r values are shown in the corresponding boxes. Red box indicates a positive correlation and blue box indicates a negative correlation. Non-significant correlations are indicated by blank. Outliers (three for CRP and two for SAA) were excluded by the defined cutoff of more than 50*interquartile range (IQR) from the median.

A.

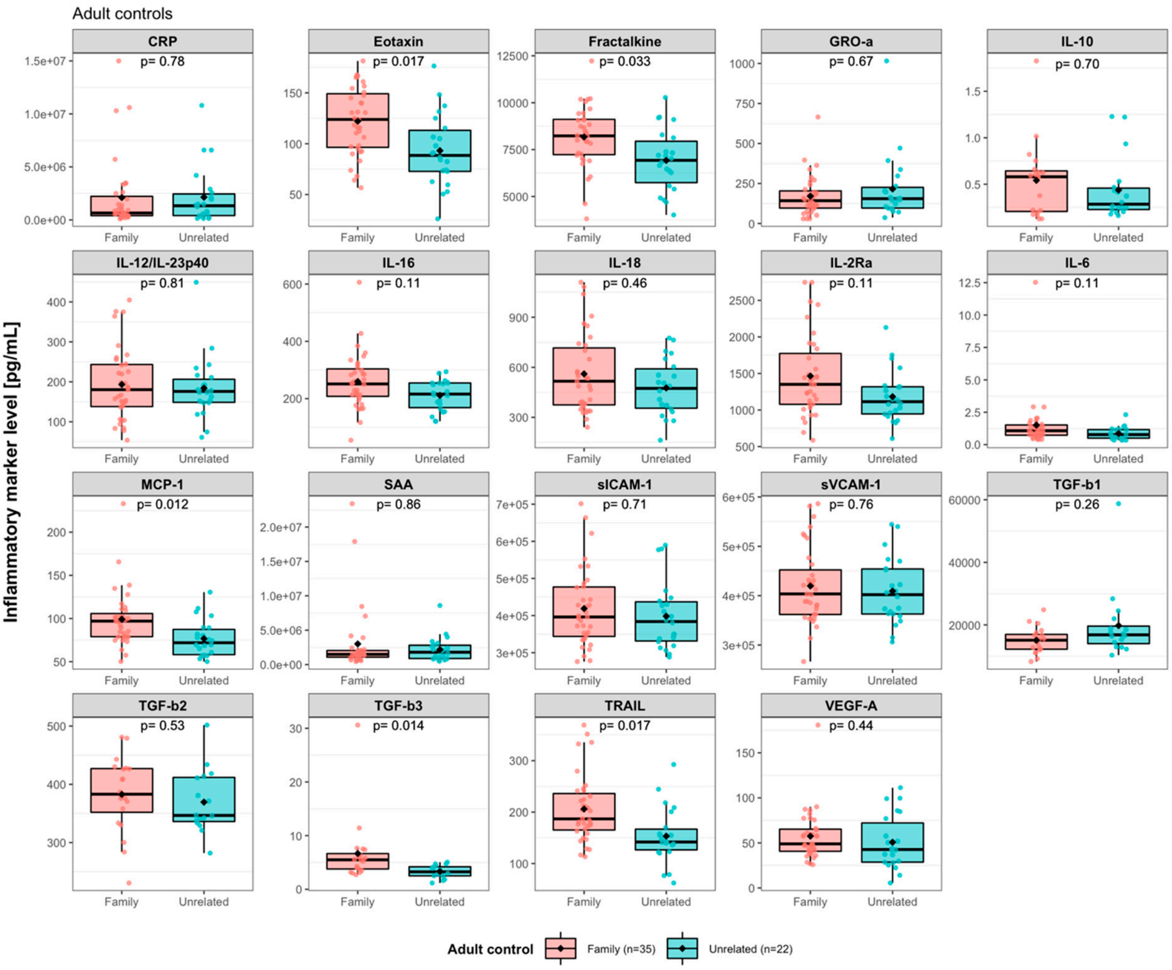
Adult controls
Inflammatory marker level [pg/mL]
CRP
p= 0.78
Eotaxin
p= 0.017
Fractalkine
p= 0.033
GRO-a
p= 0.67
IL-10
p= 0.70
IL-12/IL-23p40
p= 0.81
IL-16
p= 0.11
IL-18
p= 0.46
IL-2Ra
p= 0.11
IL-6
p= 0.11
MCP-1
p= 0.012
SAA
p= 0.86
sICAM-1
p= 0.71
sVCAM-1
p= 0.76
TGF-b1
p= 0.26
TGF-b2
p= 0.53
TGF-b3
p= 0.014
TRAIL
p= 0.017
VEGF-A
p= 0.44
Family
Unrelated
Adult control
Family (n=35)
Unrelated (n=22)

B.

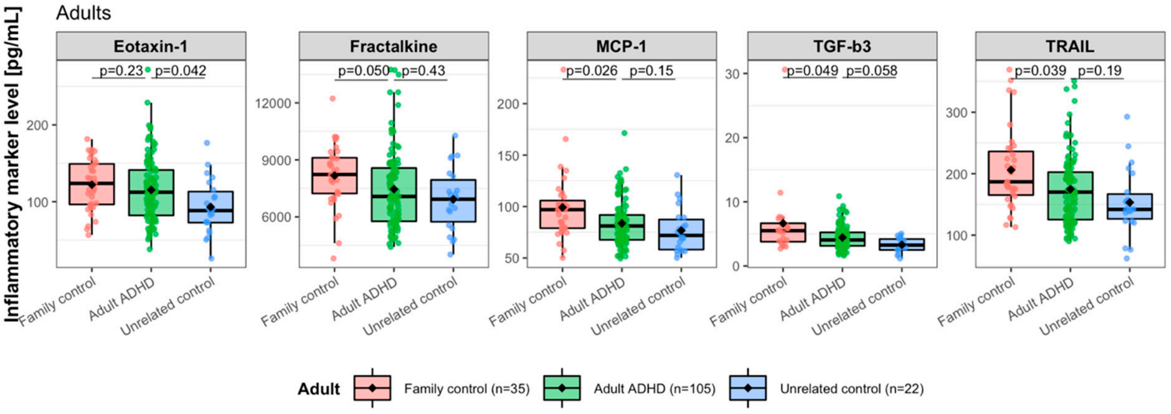
Adults
Inflammatory marker level [pg/mL]
Eotaxin-1
p=0.23
p=0.042
Fractalkine
p=0.050
p=0.43
MCP-1
p=0.026
p=0.15
TGF-b3
p=0.049
p=0.058
TRAIL
p=0.039
p=0.19
Family control
Adult ADHD
Unrelated control
Adult
Family control (n=35)
Adult ADHD (n=105)
Unrelated control (n=22)

C.

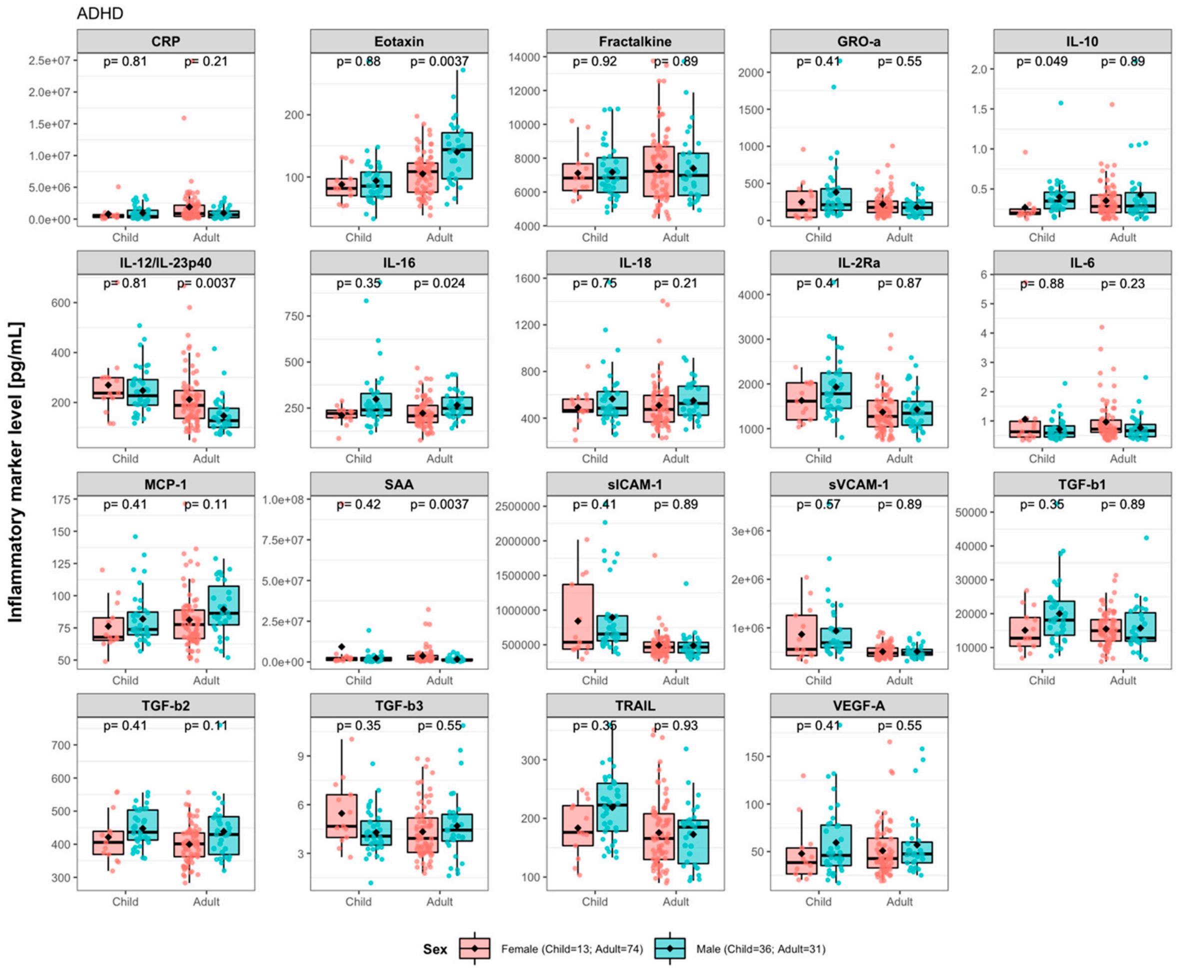
ADHD
CRP
p= 0.81
p= 0.21
Eotaxin
p= 0.88
p= 0.0037
Fractalkine
p= 0.92
p= 0.89
GRO-a
p= 0.41
p= 0.55
IL-10
p= 0.049
p= 0.89
IL-12/IL-23p40
p= 0.81
p= 0.0037
IL-16
p= 0.35
p= 0.024
IL-18
p= 0.75
p= 0.21
IL-2Ra
p= 0.41
p= 0.87
IL-6
p= 0.88
p= 0.23
MCP-1
p= 0.41
p= 0.11
SAA
p= 0.42
p= 0.0037
sICAM-1
p= 0.41
p= 0.89
sVCAM-1
p= 0.57
p= 0.89
TGF-b1
p= 0.35
p= 0.89
TGF-b2
p= 0.41
p= 0.11
TGF-b3
p= 0.35
p= 0.55
TRAIL
p= 0.36
p= 0.93
VEGF-A
p= 0.41
p= 0.55
Child
Adult
Inflammatory marker level [pg/mL]
Sex
Female (Child=13; Adult=74)
Male (Child=36; Adult=31)

D.

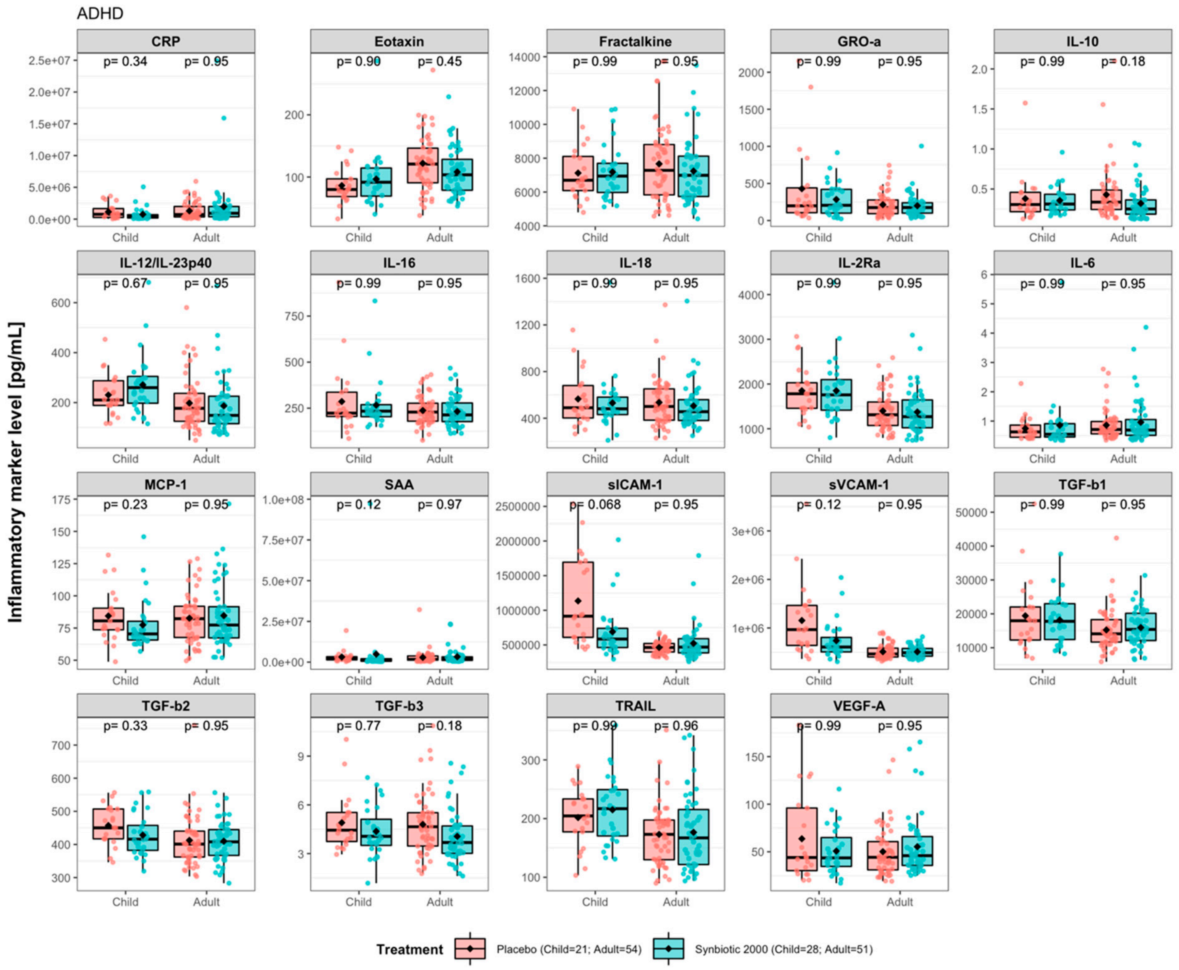
ADHD
CRP
Eotaxin
Fractalkine
GRO-a
IL-10
IL-12/IL-23p40
IL-16
IL-18
IL-2Ra
IL-6
MCP-1
SAA
sICAM-1
sVCAM-1
TGF-b1
TGF-b2
TGF-b3
TRAIL
VEGF-A
Inflammatory marker level [pg/mL]
Child
Adult
Treatment
Placebo (Child=21; Adult=54)
Synbiotic 2000 (Child=28; Adult=51)

E.

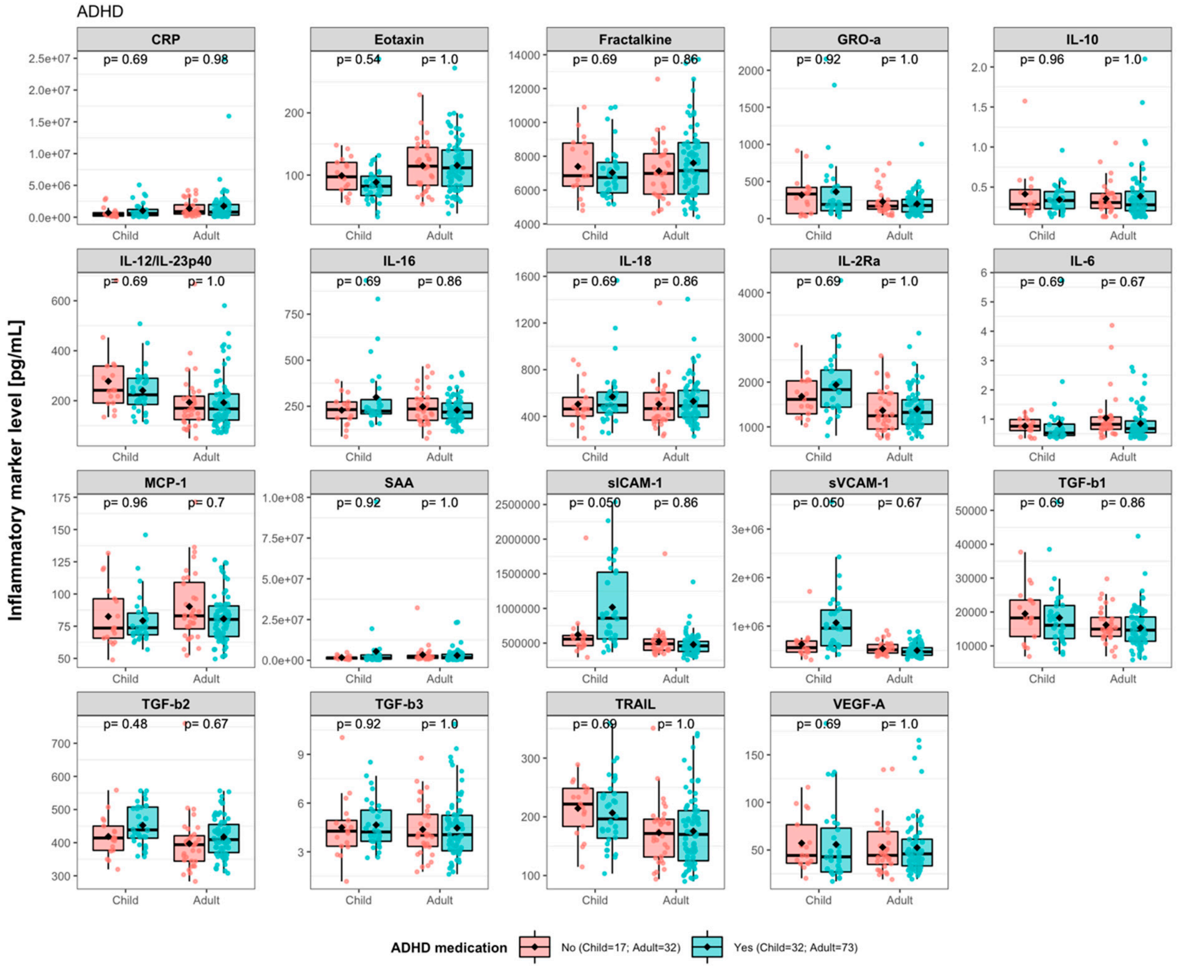

Figure S4. Immune activity marker levels <u>at baseline</u> with respect to control type, sex, treatment group and ADHD medication type stratified by age group. **(A)** Adult control types; **(B)** Adult control types and adult ADHD patients; **(C)** Sex in children and adults; **(D)** Treatment group (placebo and Synbiotic 2000) in children and adults; **(E)** ADHD medication in children and adults. Y-axes represent analyte levels. The differences between groups were analyzed using nonparametric Mann–Whitney *U* test and FDR-adjusted *p* values are shown. Each dot represents a participant. Outliers (three for CRP and two for SAA) were excluded by the defined cut-off of more than 50*interquartile range (IQR) from the median.

A.

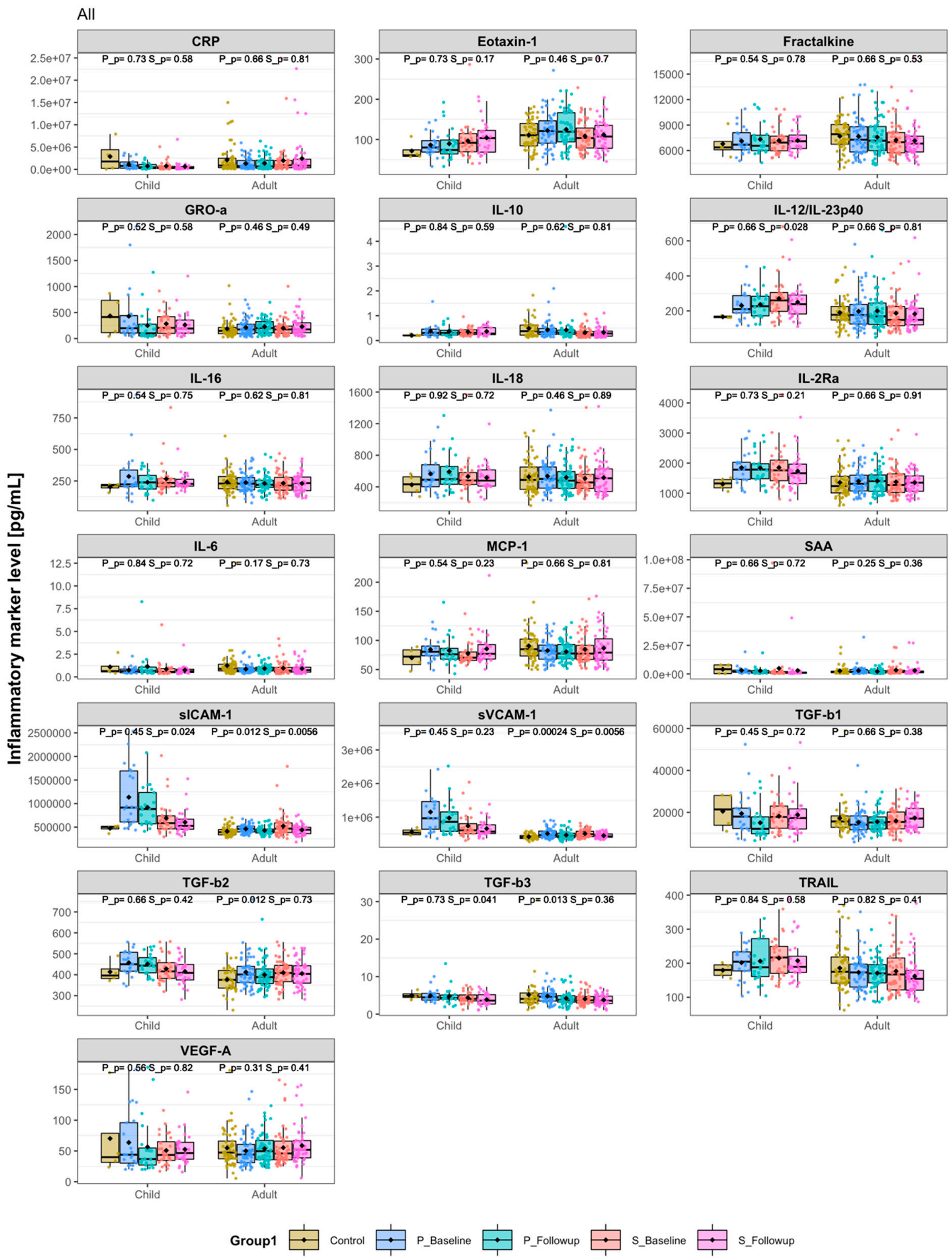

All
Inflammatory marker level [pg/mL]
CRP
P_p= 0.73 S_p= 0.58
P_p= 0.66 S_p= 0.81
Eotaxin-1
P_p= 0.73 S_p= 0.17
P_p= 0.46 S_p= 0.7
Fractalkine
P_p= 0.54 S_p= 0.78
P_p= 0.66 S_p= 0.53
GRO-a
P_p= 0.52 S_p= 0.58
P_p= 0.46 S_p= 0.49
IL-10
P_p= 0.84 S_p= 0.59
P_p= 0.62 S_p= 0.81
IL-12/IL-23p40
P_p= 0.66 S_p= 0.028
P_p= 0.66 S_p= 0.81
IL-16
P_p= 0.54 S_p= 0.75
P_p= 0.62 S_p= 0.81
IL-18
P_p= 0.92 S_p= 0.72
P_p= 0.46 S_p= 0.89
IL-2Ra
P_p= 0.73 S_p= 0.21
P_p= 0.66 S_p= 0.91
IL-6
P_p= 0.84 S_p= 0.72
P_p= 0.17 S_p= 0.73
MCP-1
P_p= 0.54 S_p= 0.23
P_p= 0.66 S_p= 0.81
SAA
P_p= 0.66 S_p= 0.72
P_p= 0.25 S_p= 0.36
sICAM-1
P_p= 0.45 S_p= 0.024
P_p= 0.012 S_p= 0.0056
sVCAM-1
P_p= 0.45 S_p= 0.23
P_p= 0.00024 S_p= 0.0056
TGF-b1
P_p= 0.45 S_p= 0.72
P_p= 0.66 S_p= 0.38
TGF-b2
P_p= 0.66 S_p= 0.42
P_p= 0.012 S_p= 0.73
TGF-b3
P_p= 0.73 S_p= 0.041
P_p= 0.013 S_p= 0.36
TRAIL
P_p= 0.84 S_p= 0.58
P_p= 0.82 S_p= 0.41
VEGF-A
P_p= 0.56 S_p= 0.82
P_p= 0.31 S_p= 0.41
Child
Adult
Group1
Control
P_Baseline
P_Followup
S_Baseline
S_Followup

B.

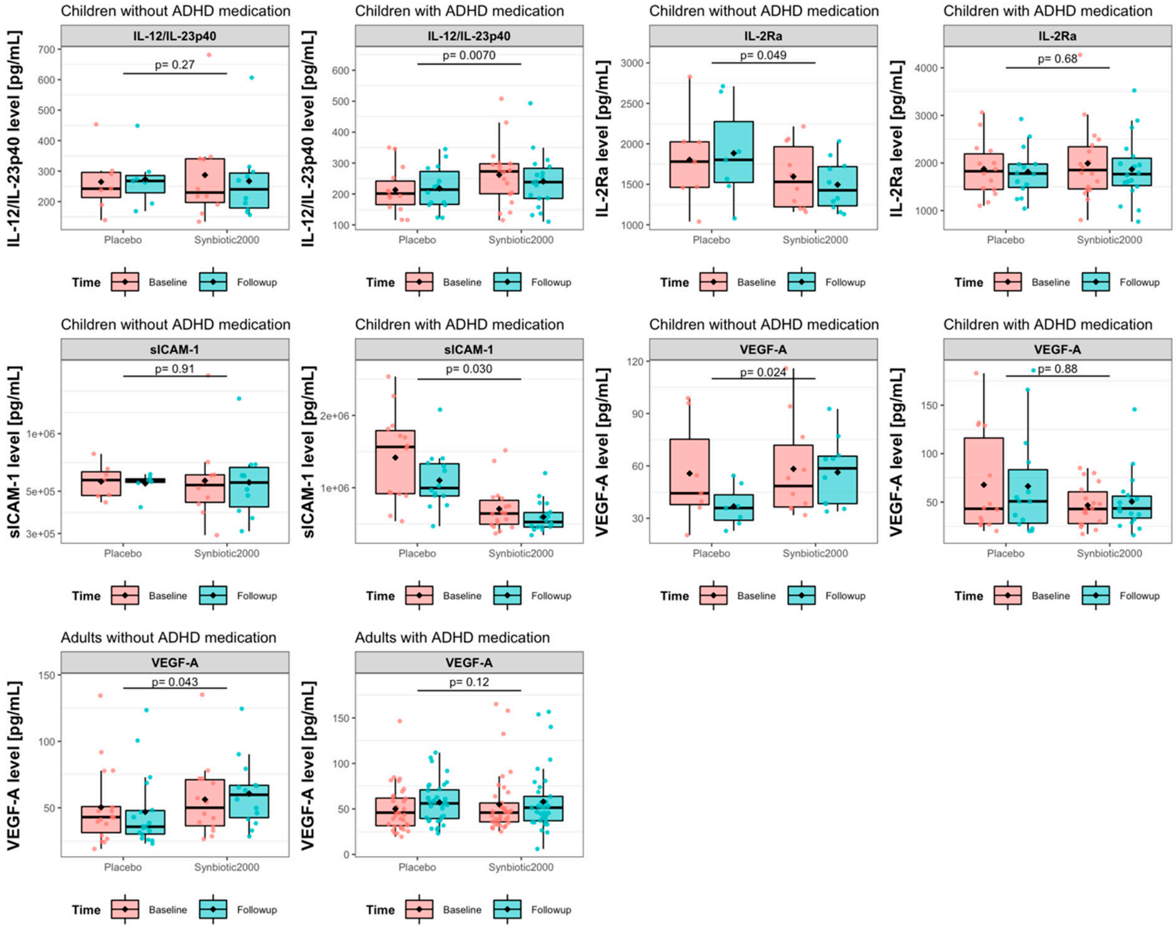

Figure S5. Plasma levels of immune activity markers: (A) Time effect (from baseline to the 9-week follow-up) for each intervention group (placebo and Synbiotic 2000); (B) Treatment effect (Synbiotic 2000 in comparison with placebo) stratified by ADHD medication. The differences from baseline to follow-up were analyzed using paired nonparametric Mann–Whitney *U* test and FDR-adjusted *p* values are shown on top. The treatment effect was determined using ANCOVA on follow-up analyte levels adjusted for sex and baseline analyte levels. “P” stands for placebo group, “S” stands for Synbiotic 2000 group. Each dot represents a participant.

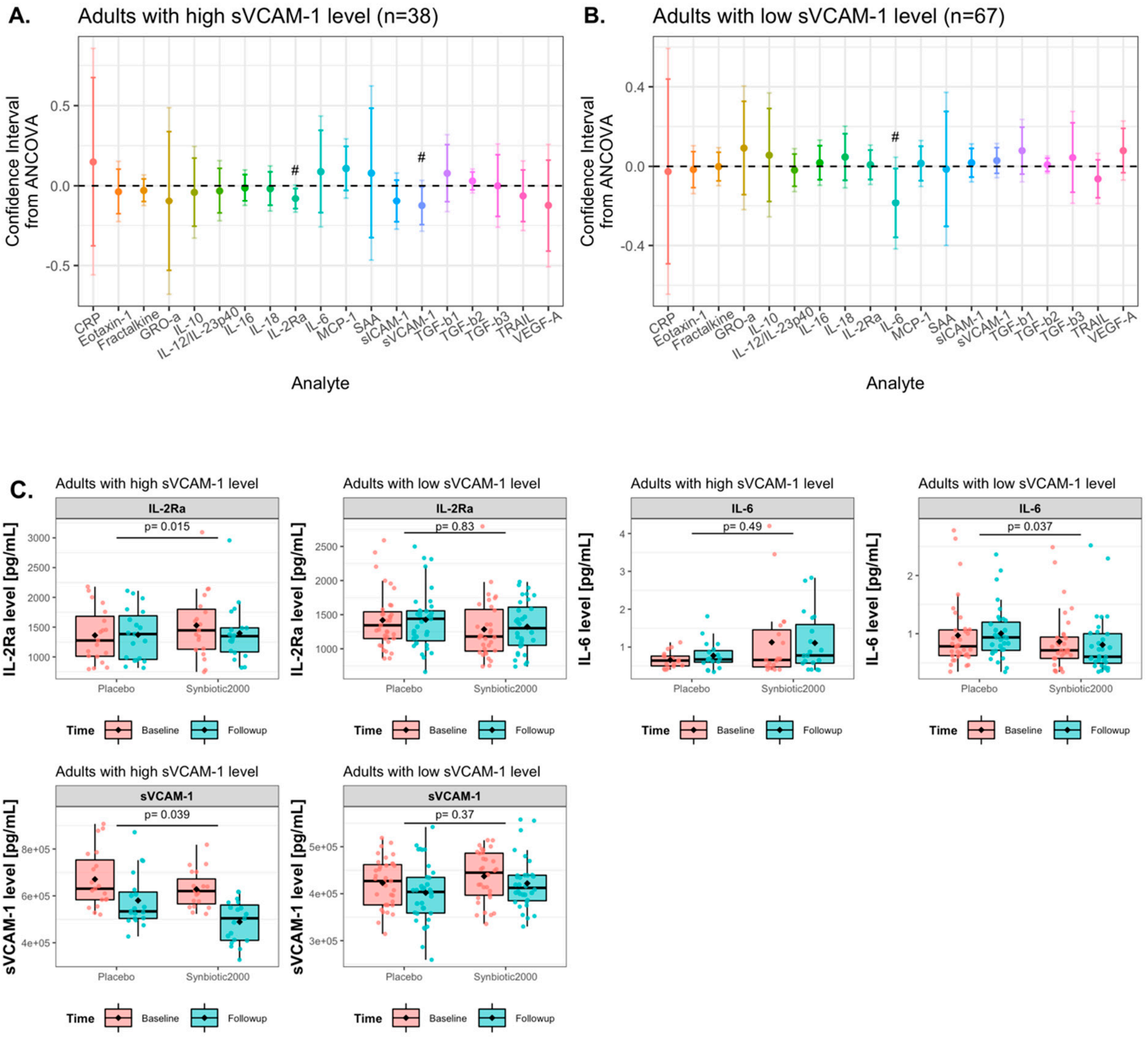

Figure S6. Plasma levels of immune activity markers: Treatment effect (Synbiotic 2000 compared to placebo) stratified by baseline plasma sVCAM-1 levels in adults with ADHD. CIs were from ANCOVA among **(A)** Adults with low sVCAM-1 level at baseline (< 519519.7 pg/mL); (**B**) Adults with high sVCAM-1 level at baseline (< 519519.7 pg/mL). Strong error bar colors indicate 95% CIs and light error bar colors indicate 99% CIs; (**C**) box plot for the analytes with Synbiotic 2000-specific effect in panel (**A**) and (**B**). A CI below 0 means that Synbiotic 2000, compared to placebo, reduced the analyte levels. The treatment effect was determined using ANCOVA on follow-up analyte levels adjusted for sex and baseline analyte levels. * Statistical significance (α=0.01); # difference at α=0.05.

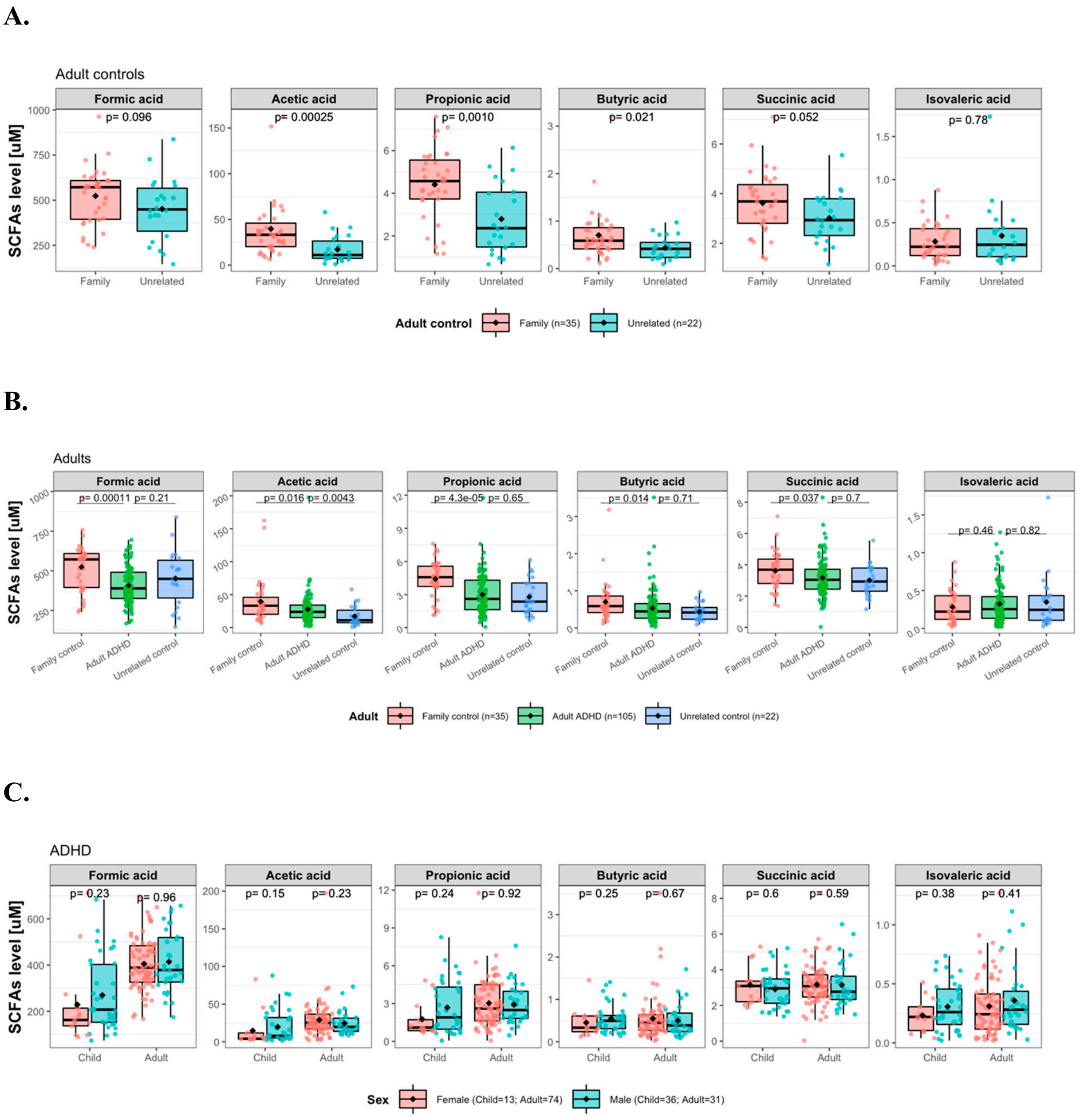
A.
Adult controls
SCFAs level [uM]
Formic acid
p= 0.096
Acetic acid
p= 0.00025
Propionic acid
p= 0.0010
Butyric acid
p= 0.021
Succinic acid
p= 0.052
Isovaleric acid
p= 0.78
Family
Unrelated
Adult control
Family (n=35)
Unrelated (n=22)
B.
Adults
SCFAs level [uM]
Formic acid
p= 0.00011
p= 0.21
Acetic acid
p= 0.016
p= 0.0043
Propionic acid
p= 4.3e-05
p= 0.65
Butyric acid
p= 0.014
p= 0.71
Succinic acid
p= 0.037
p= 0.7
Isovaleric acid
p= 0.46
p= 0.82
Family control
Adult ADHD
Unrelated control
Adult
Family control (n=35)
Adult ADHD (n=105)
Unrelated control (n=22)
C.
ADHD
SCFAs level [uM]
Formic acid
p= 0.23
p= 0.96
Acetic acid
p= 0.15
p= 0.23
Propionic acid
p= 0.24
p= 0.92
Butyric acid
p= 0.25
p= 0.67
Succinic acid
p= 0.6
p= 0.59
Isovaleric acid
p= 0.38
p= 0.41
Child
Adult
Sex
Female (Child=13; Adult=74)
Male (Child=36; Adult=31)

D.

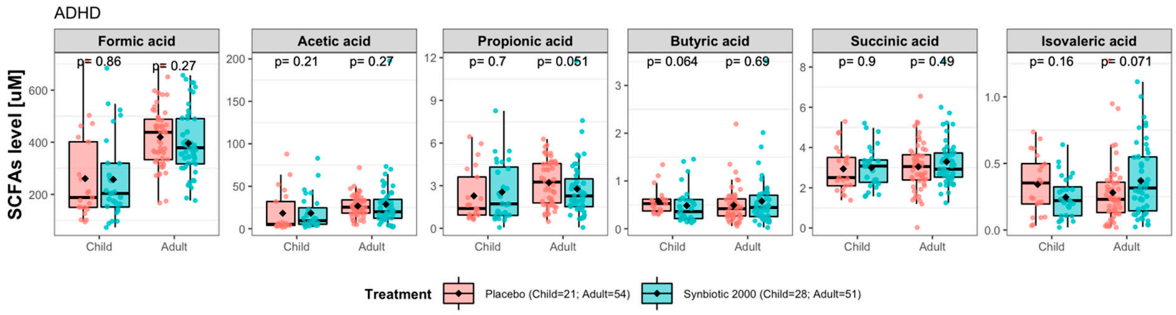

E.

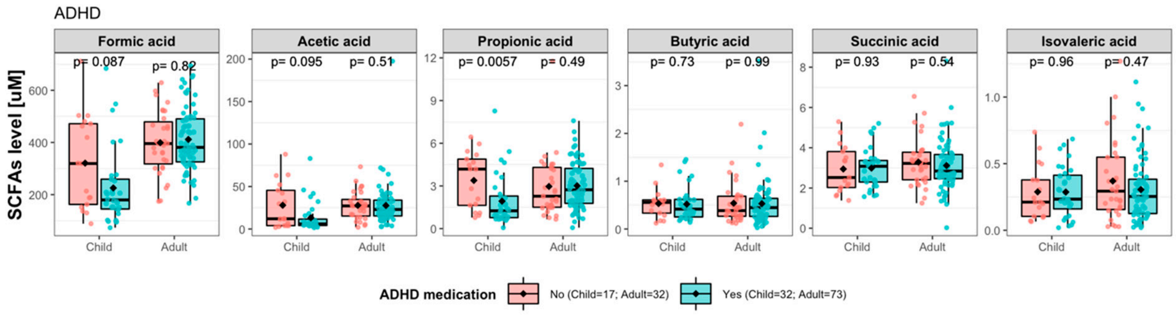

Figure S7. Short-chain fatty acid (SCFA) levels in plasma at baseline with respect to control type, sex, treatment group and ADHD medication stratified by age group. **(A)** Control type; **(B)** Diagnosis and control type; (**C**) Sex in children and adults **(D)** Treatment group in children and adults; **(E)** ADHD medication in children and adults. Y-axes represent analyte levels. The differences between groups were analyzed using nonparametric Mann–Whitney *U* test and *p* values are shown. Each dot represents a participant.

A.

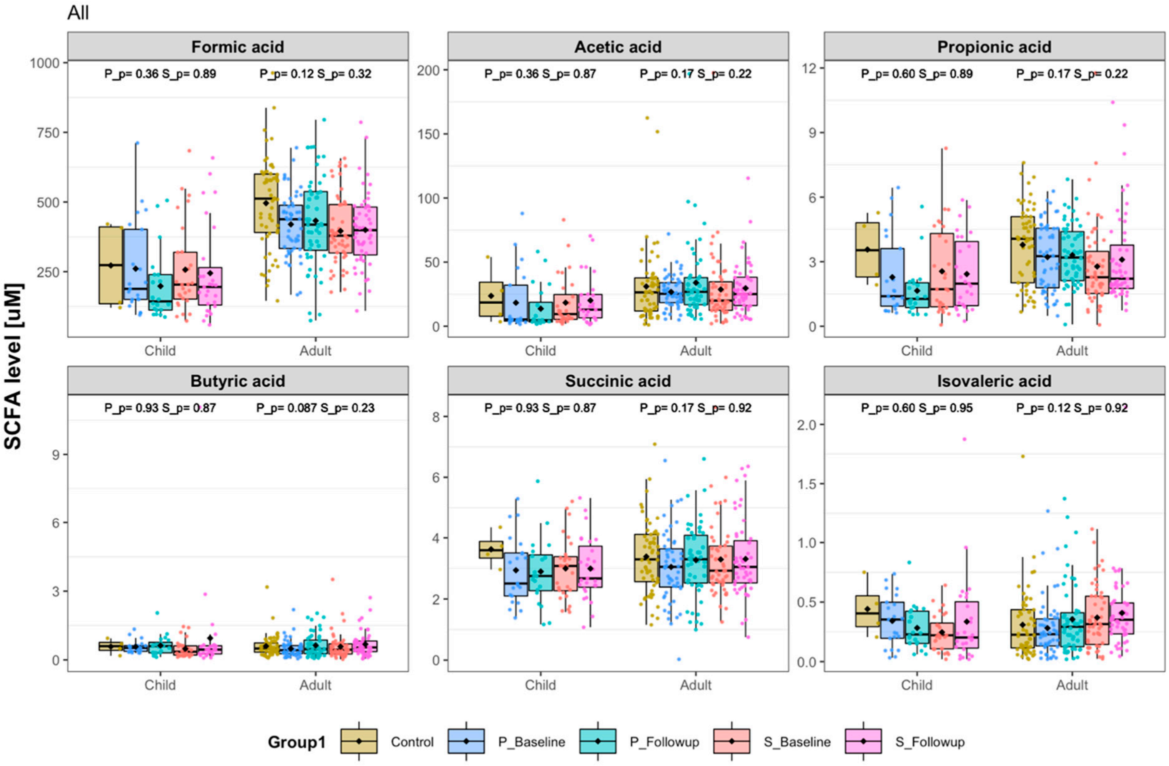

B.

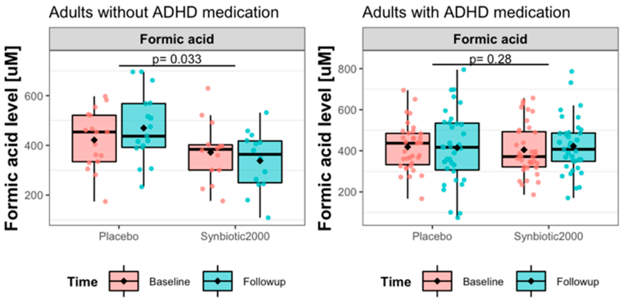

Figure S8. Plasma levels of short-chain fatty acid (SCFAs): (A) Time effect (from baseline to follow-up) on all SCFAs for each intervention group (placebo and Synbiotic 2000); (B) Treatment effect (Synbiotic 2000 in comparison with placebo) on formic acid in adults stratified by ADHD medication. The differences from baseline to follow-up were analyzed using paired nonparametric Mann–Whitney *U* test and *p* values are shown on top. The treatment effect was determined using ANCOVA on follow-up analyte levels adjusted for sex and baseline analyte levels. "P" stands for placebo group, "S" stands for Synbiotic 2000 group. Each dot represents a participant.

A. Children

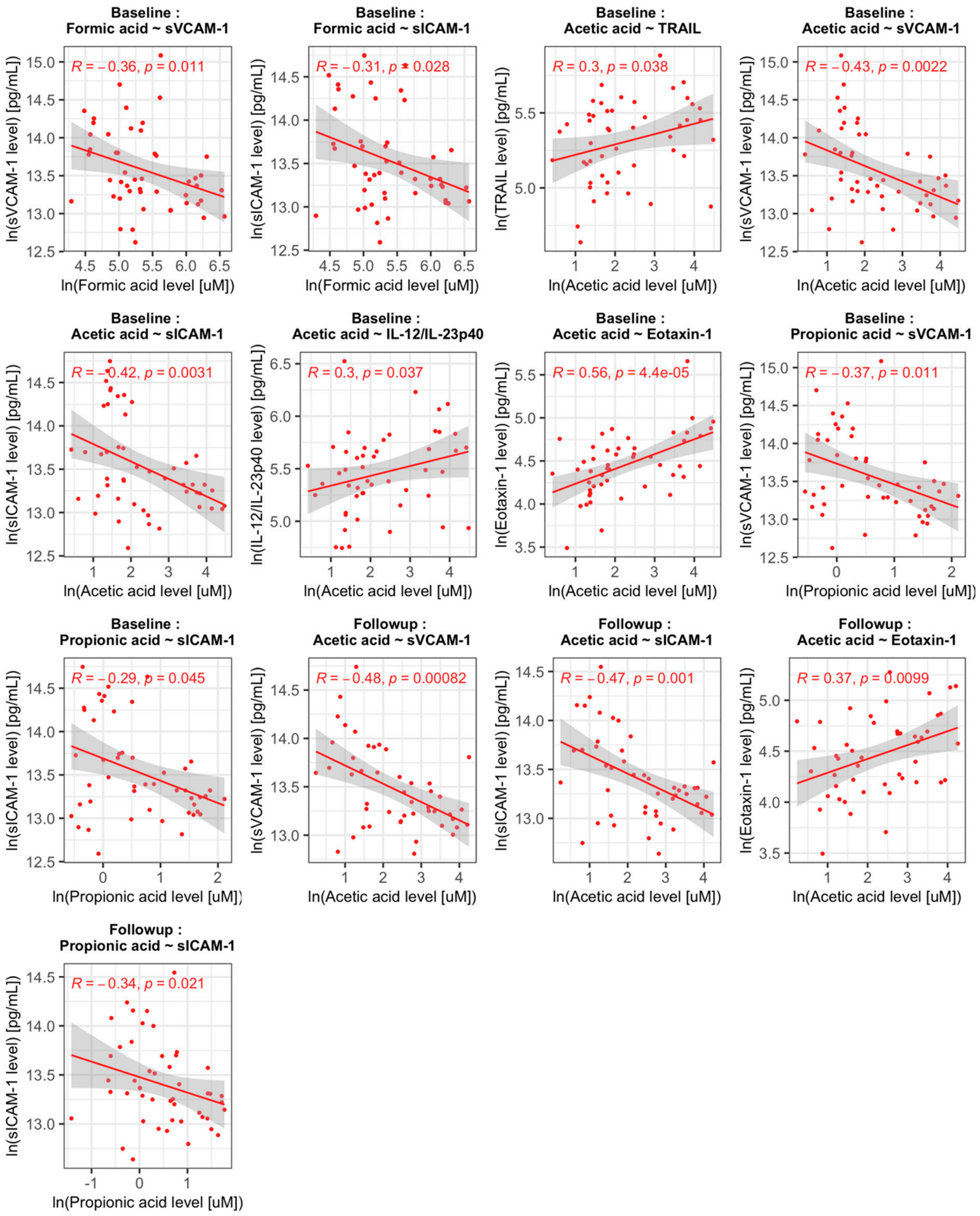

Baseline : Formic acid ~ sVCAM-1
R = −0.36, p = 0.011
ln(sVCAM-1 level) [pg/mL]
ln(Formic acid level [uM])
Baseline : Formic acid ~ sICAM-1
R = −0.31, p = 0.028
ln(sICAM-1 level) [pg/mL]
ln(Formic acid level [uM])
Baseline : Acetic acid ~ TRAIL
R = 0.3, p = 0.038
ln(TRAIL level) [pg/mL]
ln(Acetic acid level [uM])
Baseline : Acetic acid ~ sVCAM-1
R = −0.43, p = 0.0022
ln(sVCAM-1 level) [pg/mL]
ln(Acetic acid level [uM])
Baseline : Acetic acid ~ sICAM-1
R = −0.42, p = 0.0031
ln(sICAM-1 level) [pg/mL]
ln(Acetic acid level [uM])
Baseline : Acetic acid ~ IL-12/IL-23p40
R = 0.3, p = 0.037
ln(IL-12/IL-23p40 level) [pg/mL]
ln(Acetic acid level [uM])
Baseline : Acetic acid ~ Eotaxin-1
R = 0.56, p = 4.4e-05
ln(Eotaxin-1 level) [pg/mL]
ln(Acetic acid level [uM])
Baseline : Propionic acid ~ sVCAM-1
R = −0.37, p = 0.011
ln(sVCAM-1 level) [pg/mL]
ln(Propionic acid level [uM])
Baseline : Propionic acid ~ sICAM-1
R = −0.29, p = 0.045
ln(sICAM-1 level) [pg/mL]
ln(Propionic acid level [uM])
Followup : Acetic acid ~ sVCAM-1
R = −0.48, p = 0.00082
ln(sVCAM-1 level) [pg/mL]
ln(Acetic acid level [uM])
Followup : Acetic acid ~ sICAM-1
R = −0.47, p = 0.001
ln(sICAM-1 level) [pg/mL]
ln(Acetic acid level [uM])
Followup : Acetic acid ~ Eotaxin-1
R = 0.37, p = 0.0099
ln(Eotaxin-1 level) [pg/mL]
ln(Acetic acid level [uM])
Followup : Propionic acid ~ sICAM-1
R = −0.34, p = 0.021
ln(sICAM-1 level) [pg/mL]
ln(Propionic acid level [uM])

B. Adults

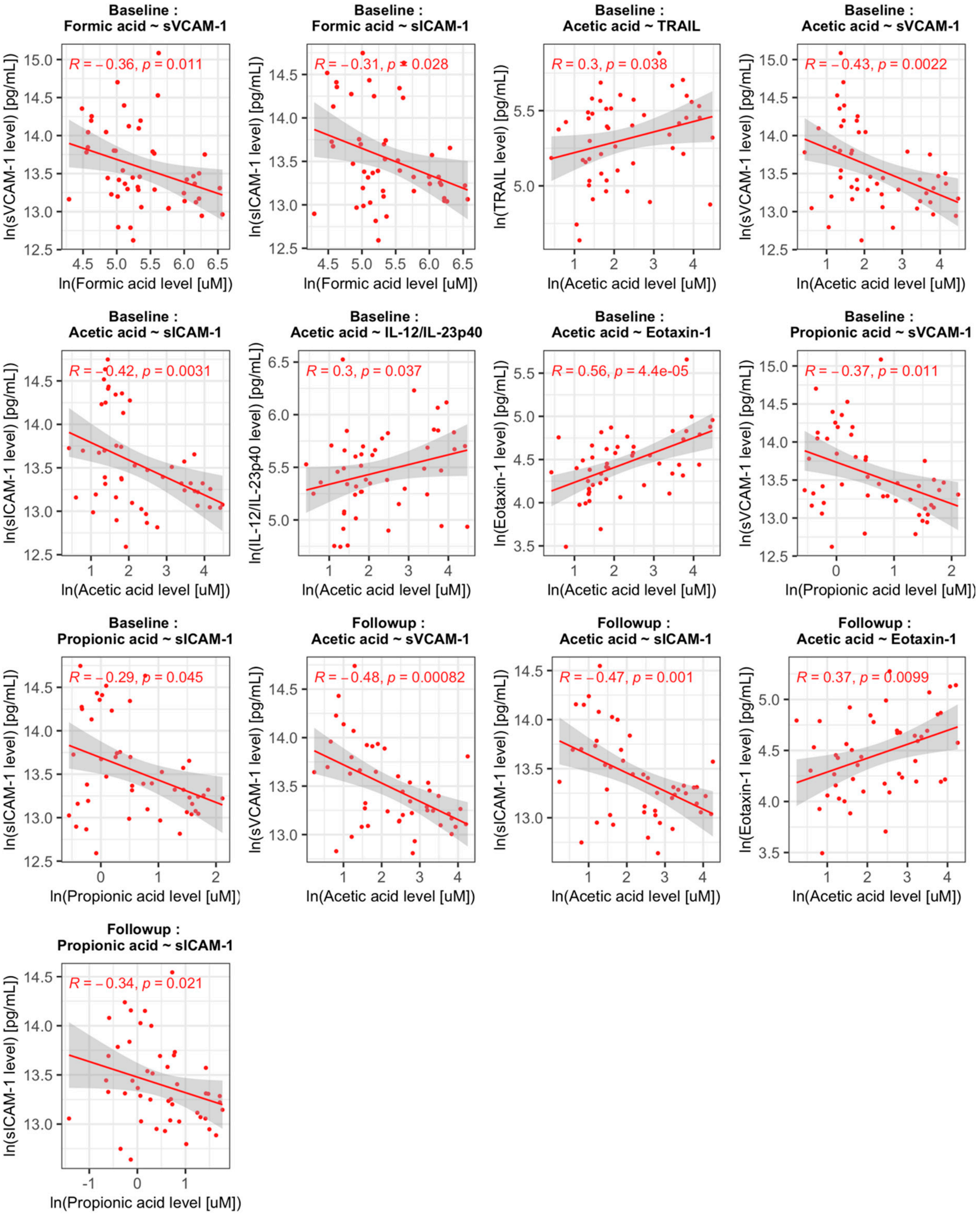

Baseline : Formic acid ~ sVCAM-1
R = –0.36, p = 0.011
ln(sVCAM-1 level) [pg/mL]
ln(Formic acid level [uM])
Baseline : Formic acid ~ sICAM-1
R = –0.31, p = 0.028
ln(sICAM-1 level) [pg/mL]
ln(Formic acid level [uM])
Baseline : Acetic acid ~ TRAIL
R = 0.3, p = 0.038
ln(TRAIL level) [pg/mL]
ln(Acetic acid level [uM])
Baseline : Acetic acid ~ sVCAM-1
R = –0.43, p = 0.0022
ln(sVCAM-1 level) [pg/mL]
ln(Acetic acid level [uM])
Baseline : Acetic acid ~ sICAM-1
R = –0.42, p = 0.0031
ln(sICAM-1 level) [pg/mL]
ln(Acetic acid level [uM])
Baseline : Acetic acid ~ IL-12/IL-23p40
R = 0.3, p = 0.037
ln(IL-12/IL-23p40 level) [pg/mL]
ln(Acetic acid level [uM])
Baseline : Acetic acid ~ Eotaxin-1
R = 0.56, p = 4.4e-05
ln(Eotaxin-1 level) [pg/mL]
ln(Acetic acid level [uM])
Baseline : Propionic acid ~ sVCAM-1
R = –0.37, p = 0.011
ln(sVCAM-1 level) [pg/mL]
ln(Propionic acid level [uM])
Baseline : Propionic acid ~ sICAM-1
R = –0.29, p = 0.045
ln(sICAM-1 level) [pg/mL]
ln(Propionic acid level [uM])
Followup : Acetic acid ~ sVCAM-1
R = –0.48, p = 0.00082
ln(sVCAM-1 level) [pg/mL]
ln(Acetic acid level [uM])
Followup : Acetic acid ~ sICAM-1
R = –0.47, p = 0.001
ln(sICAM-1 level) [pg/mL]
ln(Acetic acid level [uM])
Followup : Acetic acid ~ Eotaxin-1
R = 0.37, p = 0.0099
ln(Eotaxin-1 level) [pg/mL]
ln(Acetic acid level [uM])
Followup : Propionic acid ~ sICAM-1
R = –0.34, p = 0.021
ln(sICAM-1 level) [pg/mL]
ln(Propionic acid level [uM])

Figure S9. Individual scatter plots of the correlations between plasma levels of immune activity markers and short-chain fatty acids (SCFAs) shown in Fig. 6 with un-adjusted *p* < 0.05. (A) Children at baseline and follow-up; **(B)** Adults at baseline and follow-up. Spearman's correlation analysis with natural logarithm (ln)-transformed analyte levels. *r* and un-adjusted *p* values are shown. Outliers (three for CRP and two for SAA) were excluded by the defined cut-off of more than 50*interquartile range (IQR) from the median.

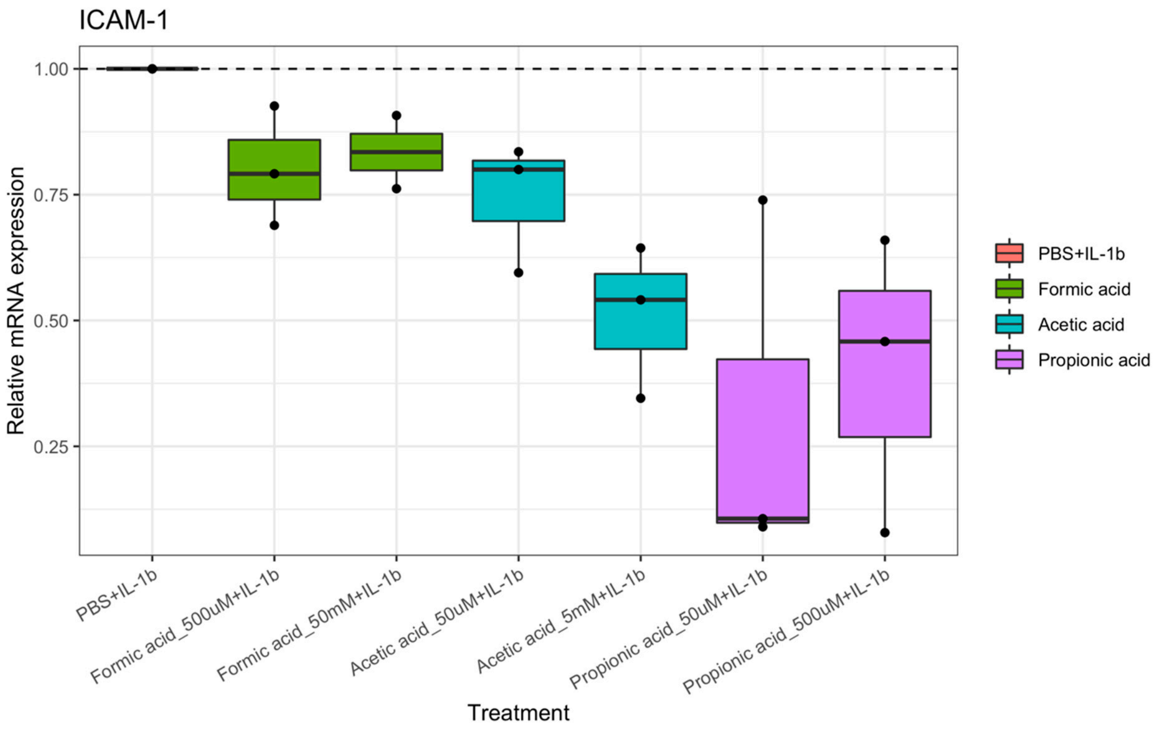

Figure S10. IL-1*β*-induced ICAM-1 mRNA expression in human aortic vascular smooth-muscle-cells after *in vitro* pre-incubation with different short-chain fatty acids (SCFAs). The results from 3 independent experiments (biological replicas) are shown.

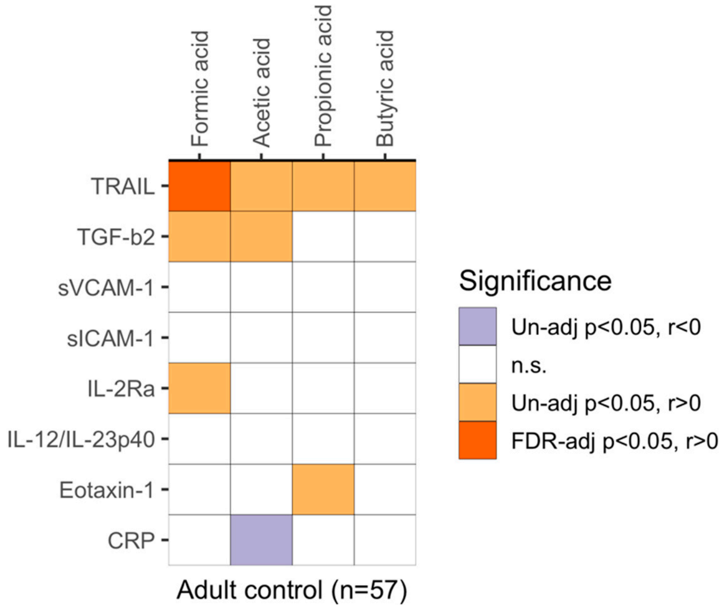

Figure S11. Correlation analysis between plasma levels of immune activity markers and short-chain fatty acids (SCFAs) in adult controls. Spearman's rank correlation coefficients were calculated. FDR-adjusted significant correlations (p<0.05) are indicated in strong orange colour, and unadjusted correlations (p<0.05) are indicated in light orange or light blue colour. Orange indicates a positive correlation, and blue a negative correlation.

Table S1. *Experimental details of 24 immune activity analytes measured using Meso Scale Discovery in 61 controls and 154 ADHD patients.*

Analyte	Other name/full name	Lower limit of detection (LLOD) pg/mL (n=5)	Between-plate CV (%)	Within-plate CV (%)	Number of values below LLOD: total (control/baseline/follow-up)[a]
CRP	C-reactive protein	2289-3350	4.63	2.39	0 (0/0/0)
Eotaxin-1		12.70-21.85	10.8	3.13	0 (0/0/0)
Fractalkine	CX3CL1 (C-X3-C motif chemokine ligand 1)	80.5-259.1	16.9	2.23	0 (0/0/0)
GRO-α	CXCL1 (C-X-C motif chemokine ligand 1)	0.37-0.71	11.9	1.86	0 (0/0/0)
IL-10	CSIF (cytokine synthesis inhibitory factor)/interleukin 10	0.13-0.56	7.13	3.78	56 (22/18/16)
IL-12/IL-23p40	interleukin-12 subunit p40 or β	2.25-4.14	12.3	3.48	0 (0/0/0)
IL-16	interleukin-16	6.79-16.17	10.7	1.10	0 (0/0/0)
IL-18	interleukin-18	0.57-1.52	10.2	1.52	0 (0/0/0)
IL-2Rα	interleukin-2 receptor α	8.23-16.78	10.1	2.54	0 (0/0/0)
IL-6	interleukin-16	0.34-0.73	8.60	2.41	52 (11/20/21)
MCP-1	CCL2 (C-C motif chemokine ligand 2)/monocyte chemoattractant protein 1	0.72-1.53	9.93	2.00	0 (0/0/0)
SAA	serum amyloid A	7999-13025	11.0	1.48	0 (0/0/0)
sICAM-1	soluble cell adhesion molecule 1	997.4-1610.0	8.93	5.28	0 (0/0/0)
sVCAM-1	soluble vascular cell adhesion molecule 1	5429-8426	6.59	1.63	0 (0/0/0)
TGF-β1	transforming growth factor β 1	6.70-46.36	8.67	4.90	0 (0/0/0)
TGF-β2	transforming growth factor β 2	2.20-10.54	6.60	3.45	0 (0/0/0)
TGF-β3	transforming growth factor β 3	1.05-1.95	7.91	4.70	8 (1/1/6)
TRAIL	TNF-related apoptosis-inducing ligand	0.83-2.66	11.02	2.60	0 (0/0/0)
VEGF-A	vascular endothelial growth factor A	3.91-6.05	6.25	3.80	1 (0/0/1)
INF-γ[b]	interferon γ	4.84-15.94	25.21	6.49	166 (25/74/67)
IL-1β[b]	interleukin 1 β	0.20-0.45	8.61	2.08	308 (47/128/133)
IL-17A[b]	interleukin 17A	1.77-4.57	5.21	6.53	247 (38/15/104)
IL-2[b]	interleukin 2	0.98-2.37	5.70	4.16	334 (54/140/140)
TNF-α[b]	tumor necrosis factor α	0.59-3.70	8.71	2.71	103 (33/28/42)

CV: coefficient of variation.

a. The values below LLOD were replaced with LLOD in the data analysis;

b. These five analytes with more 25% values below LLOD were excluded from data analysis;

Table S2 *Confidence intervals for treatment effects shown in* ***Fig 3, Fig 5, Fig S5B, Fig S6, Fig S8B.***

**Analyte**	**Age**	**Group**	**95% CI**	**99% CI**	***p* value**
IL-12/IL-23p40	Children	All	-0.158, -0.014	-0.182, 0.010	0.020
IL-12/IL-23p40	Children	With ADHD medication	-0.157, -0.028	-0.180, -0.005	0.0070*
sICAM-1	Children	With ADHD medication	-0.547, -0.030	-0.637, 0.060	0.030
sIL-2Ra	Children	Without ADHD medication	-0.274, -0.001	-0.328, 0.053	0.049
VEGF-A	Children	Without ADHD medication	0.054, 0.644	-0.062, 0.760	0.024
VEGF-A	Adults	Without ADHD medication	0.007, 0.368	-0.056, 0.431	0.043
sIL-2Ra	Adults	With high sVCAM-1	-0.145, -0.017	-0.167, 0.005	0.015
IL-6	Adults	With low sVCAM-1	-0.359, -0.011	-0.417, 0.046	0.037
sVCAM-1	Adults	With high sVCAM-1	-0.245, -0.007	-0.286, 0.034	0.039
Propionic acid	Children	All	0.006, 0.699	-0.110, 0.816	0.046
Formic acid	Adults	Without ADHD medication	-0.522, -0.024	-0.609, 0.063	0.033

*p <0.01; CI: confidence interval;

Table S3. *Clinical characteristics of adult participants at baseline.*

		ADHD patients[e] (n=105)	Controls (n=57)
		**Median (IQR)/N (%)**	**Median (IQR)/N (%)**
Age [years]		36 (29-43)	38 (34-43)
Body mass index [kg/m^2]		23.8 (22.2-26.6)	23.6 (21.6-27.6)
Sex	Female	74 (70)	36 (63)
	Male	31 (30)	21 (37)
Antibiotic drugs[a]	≥1 time	30 (28)	11 (20)
	None	70 (67)	46 (81)
	Unknown	5 (5)	0 (0)
Melatonin medication	Yes†	26 (25)	0 (0)*
	No†	79 (75)	57 (100)
ADHD medication[b]	Yes†	73 (70)	0 (0)*
	No†	32 (30)	57 (100)
Other prescribed drug for adults[c]	Yes†	54 (51)	3 (5)*
	No†	51 (49)	54 (95)
Dietary supplements[d]	Yes	76 (72)	27 (47)*
	No	29 (28)	30 (53)

Results are given as median (25th-75th percentile [IQR]) or as number (%) of subjects; **a**. number of antibiotic drug uses in the last two years (no one was on antibiotic drug use last 6 weeks); **b**. ADHD medications for children include the stimulants Methylphenidate (n=14), Lisdexamphetamine (n=10), the nonstimulant Atomoxetine (noradrenalin re-uptake inhibitor (n=4) and Methylphenidate plus Atomoxetine (n=3), and for adults they include Methylphenidate (n=34), Lisdexamphetamine (n=34), Dexamphetamine (n=12), Atomoxetine (n=3) and Methylphenidate plus Atomoxetine (n=1); **c**. other prescribed drugs for adults reported to influence immune activity and gut microbiome include Antidepressants, Antipsychotics, Anxiolytics, Sleeping pills (mainly antihistamines), Proton-pump inhibitors and Statins; **d**. supplements (e.g. vitamins, omega-3, probiotics) taken in the last 4 weeks. The probiotics used were *L. plantarum 299v* (1 control, 11 patients), Synbiotic 15 (similar constituents as Synbiotic 2000 but $15*10^9$ CFU instead of the $4*10^{11}$CFU in Synbiotic 2000, 2 patients) and other (6 patients). **e**. ADHD ICD-10 diagnosis: F90.0 (18.8%), F90.0B (57.8%), F90.0C (17.5%), F90.0X (2.6%), F90.1 (0.7%), F90.8 (0.7%), F98.8 (2.0%); † Participants on medication currently or in last 3 months as "Yes", not on medication currently or in the last 3 months as "No". * Difference between adult ADHD patients and adult controls at α=0.05.

Note for ADHD medication:

Medication data was collected including medication for core ADHD symptoms (here called ADHD medication), antibiotic drugs, and other medications previously reported to influence immune activity or gut microbiome i.e., anti-inflammatory melatonin (Carrascal et al., 2018; Tarocco et al., 2019), antidepressants (Macedo et al., 2017; Więdłocha et al., 2018), antipsychotics (Bobermin et al., 2018), anxiolytics (Bretler et al., 2019; Chen et al., 2018; Lazzarini et al., 2001), sleeping pills (Tiligada et al., 2011), proton-pump inhibitors (Kanagaratham et al., 2020) and statins (Pinal-Fernandez et al., 2018).

References:

Bobermin, L.D., da Silva, A., Souza, D.O., Quincozes-Santos, A., 2018. Differential effects of typical and atypical antipsychotics on astroglial cells in vitro. International journal of developmental neuroscience : the official journal of the International Society for Developmental Neuroscience 69, 1-9.

Bretler, T., Weisberg, H., Koren, O., Neuman, H., 2019. The effects of antipsychotic medications on microbiome and weight gain in children and adolescents. BMC medicine 17, 112.

Carrascal, L., Nunez-Abades, P., Ayala, A., Cano, M., 2018. Role of Melatonin in the Inflammatory Process and its Therapeutic Potential. Current pharmaceutical design 24, 1563-1588.

Chen, C.H., Shyue, S.K., Hsu, C.P., Lee, T.S., 2018. Atypical Antipsychotic Drug Olanzapine Deregulates Hepatic Lipid Metabolism and Aortic Inflammation and Aggravates Atherosclerosis. Cell Physiol Biochem 50, 1216-1229.

Kanagaratham, C., El Ansari, Y.S., Sallis, B.F., Hollister, B.A., Lewis, O.L., Minnicozzi, S.C., Oyoshi, M.K., Rosen, R., Nurko, S., Fiebiger, E., Oettgen, H.C., 2020. Omeprazole inhibits IgE-mediated mast cell activation and allergic inflammation induced by ingested allergen in mice. The Journal of allergy and clinical immunology.

Lazzarini, R., Malucelli, B.E., Palermo-Neto, J., 2001. Reduction of acute inflammation in rats by diazepam: role of peripheral benzodiazepine receptors and corticosterone. Immunopharmacology and immunotoxicology 23, 253-265.

Macedo, D., Filho, A., Soares de Sousa, C.N., Quevedo, J., Barichello, T., Júnior, H.V.N., Freitas de Lucena, D., 2017. Antidepressants, antimicrobials or both? Gut microbiota dysbiosis in depression and possible implications of the antimicrobial effects of antidepressant drugs for antidepressant effectiveness. Journal of affective disorders 208, 22-32.

Pinal-Fernandez, I., Casal-Dominguez, M., Mammen, A.L., 2018. Statins: pros and cons. Medicina clinica 150, 398-402.

Tarocco, A., Caroccia, N., Morciano, G., Wieckowski, M.R., Ancora, G., Garani, G., Pinton, P., 2019. Melatonin as a master regulator of cell death and inflammation: molecular mechanisms and clinical implications for newborn care. Cell death & disease 10, 317.

Tiligada, E., Kyriakidis, K., Chazot, P.L., Passani, M.B., 2011. Histamine pharmacology and new CNS drug targets. CNS neuroscience & therapeutics 17, 620-628.

Więdłocha, M., Marcinowicz, P., Krupa, R., Janoska-Jaździk, M., Janus, M., Dębowska, W., Mosiołek, A., Waszkiewicz, N., Szulc, A., 2018. Effect of antidepressant treatment on peripheral inflammation markers - A meta-analysis. Progress in neuro-psychopharmacology & biological psychiatry 80, 217-226.

Table S4 *Time effects over 9 weeks on inflammatory markers.*

		Placebo				Synbiotic 2000			
**Age group**	**Analyte [pg/mL]**	**t1 median (IQR)**	**t2 median (IQR)**	**FDR-adjusted p_{time}**	**Estimate of t1-t2**	**t1 median (IQR)**	**t2 median (IQR)**	**FDR-adjusted p_{time}**	**Eatimate of t1-t2**
**Child**	CRP	745053.3 (259255.4, 1346089.8)	435575 (156108.6, 1075754.4)	0.73	118080	355349.9 (179620.8, 697018.5)	279970 (189909.7, 642704.4)	0.58	82026.1
	eotaxin-1	79.9 (68.6, 97.1)	73.9 (64, 98.7)	0.73	2.2	91.6 (69.4, 114.5)	103.5 (68.4, 122.6)	0.17	-7.0
	Fractalkine	6697.4 (6084.7, 8104.7)	6560 (5979.4, 7806.4)	0.54	-304.4	6941.7 (5972, 7697.6)	7093.3 (6239.9, 7820.6)	0.78	-73.1
	GRO-a	198.4 (105.5, 440.2)	99.3 (49.9, 277.8)	0.52	84.8	205.6 (99.5, 420.6)	192.4 (103.3, 354.9)	0.54	27.8
	IL-10	0.3 (0.2, 0.5)	0.3 (0.2, 0.4)	0.84	0	0.3 (0.2, 0.4)	0.3 (0.2, 0.5)	0.59	0.02
	IL-12/IL-23p40	210.2 (188.6, 287.4)	226.3 (171.5, 283.3)	0.66	-5.1	259.8 (197.4, 305)	238.3 (182.3, 290.6)	**0.028***	21.4
	IL-16	223.1 (205.2, 336.8)	238.3 (185.7, 294.4)	0.54	15.3	233.9 (203.7, 269.1)	226.7 (209, 263.1)	0.75	4.2
	IL-18	489.6 (402.1, 680)	498.7 (432.2, 657.7)	0.92	-2.4	480.2 (430.9, 580.9)	479 (409.8, 615)	0.72	7.2
	IL-2Ra	1782.6 (1457.7, 2028.4)	1786.1 (1479.4, 1979.3)	0.73	36.6	1758.1 (1415.8, 2099.3)	1662.7 (1317.5, 1963.7)	0.21	88.2
	IL-6	0.6 (0.4, 0.9)	0.6 (0.5, 1.1)	0.84	0	0.5 (0.5, 0.9)	0.6 (0.5, 0.8)	0.72	0.03
	MCP-1	80.5 (73.6, 90.4)	76.2 (67.7, 86.4)	0.54	5.9	70.4 (65.7, 80.3)	77 (67.9, 92.8)	0.23	-5.3
	SAA	1646595.2 (1252895.4, 3287193)	1161097.9 (732673.6, 2960056.1)	0.66	266012	970279.5 (585854.1, 2078620.7)	1074418.8 (582429.5, 1542974.5)	0.72	112153
	sICAM-1	914517.1 (610039.9, 1694365.8)	884480.1 (570049.7, 1234924.3)	0.45	185610	584787.8 (462437.7, 739473.6)	526133 (454646.4, 669398.4)	**0.024***	46776.6
	sVCAM-1	965338.4 (638909.5, 1463139.7)	859344.7 (576357.4, 1154789.6)	0.45	104628	606946.4 (506038.6, 804800.4)	566870.8 (509538.5, 769932.6)	0.23	49783.8
	TGF-b1	17959.4 (12277.6, 22037.2)	12163.9 (9785.9, 17870.5)	0.45	4057.6	17794.4 (12419.7, 22992.3)	17284.7 (12061.5, 21661.2)	0.72	816.9
	TGF-b2	450.1 (416.9, 507)	443.1 (409.2, 481.7)	0.66	7.2	416.1 (382.2, 457.6)	408.8 (375.1, 449.1)	0.42	13.3
	TGF-b3	4.4 (3.7, 5.5)	4.3 (4, 5.1)	0.73	0.2	4.1 (3.5, 5.1)	3.6 (2.7, 5.2)	**0.041***	0.6
	TRAIL	204.5 (177.4, 233.5)	188.2 (160.8, 272.2)	0.84	3.2	216.9 (170.4, 249.3)	189.6 (173, 220.5)	0.54	8.6
	VEGF-A	44 (30.2, 96)	36.9 (27.1, 54.9)	0.56	4.8	43.6 (34.6, 65)	46.7 (36.6, 64)	0.82	-1.0

**Adult**	CRP	721280.6 (347931.2, 1994883.4)	614275.8 (257305.5, 2054455.8)	0.66	-30546	923019.9 (365711.5, 1992913)	776434.2 (333025.4, 2016611.4)	0.81	36493.6
	eotaxin-1	120.9 (90.8, 146.4)	119.4 (93.9, 166.6)	0.45	-4.1	103.5 (78.8, 128.4)	107.3 (78.1, 135.1)	0.70	-2.5
	Fractalkine	7282.6 (5832.9, 8806.8)	7156.8 (5988.6, 8832.5)	0.66	47.6	6986 (5735.2, 8121.7)	6818 (5833.8, 7694.5)	0.53	137.1
	GRO-a	174.4 (92.6, 277)	201.4 (109.7, 326)	0.46	-13.4	175.1 (99.5, 239.5)	175.7 (112.3, 303.1)	0.49	-15.6
	IL-10	0.3 (0.2, 0.5)	0.3 (0.3, 0.4)	0.62	0	0.3 (0.2, 0.4)	0.3 (0.2, 0.4)	0.81	-0.004
	IL-12/IL-23p40	176.8 (125, 236.9)	169.1 (122.7, 245.1)	0.66	-2.5	148.6 (115.3, 225.4)	148.7 (120.3, 215)	0.81	1.4
	IL-16	228.8 (179.5, 276.9)	219.1 (198.3, 256.1)	0.62	6	213.5 (177.4, 278.3)	232.8 (172.8, 280.9)	0.81	1.7
	IL-18	500.8 (383.2, 651.6)	478.8 (380.6, 593.6)	0.46	14.6	455.7 (380.2, 559.1)	508.6 (340.8, 628.1)	0.89	1.9
	IL-2Ra	1313.5 (1073.3, 1598.1)	1400.2 (1060.2, 1605.4)	0.66	-14	1270.2 (1022.7, 1644.6)	1348.7 (1062.7, 1575.2)	0.91	4.2
	IL-6	0.7 (0.6, 1)	0.8 (0.7, 1.1)	0.17	-0.1	0.7 (0.5, 1.1)	0.7 (0.5, 1.1)	0.73	0.03
	MCP-1	82.2 (67.7, 91.9)	77.6 (68.5, 93)	0.66	0.9	77.3 (67.3, 91.6)	79.1 (66.2, 102.8)	0.81	-1.0
	SAA	1703542 (1228098.7, 3789533.2)	1245891.6 (772594.2, 2850467.6)	0.25	253102	2009562.8 (1071442.7, 3302860.7)	1426810.9 (1027341, 2502272.2)	0.36	285248
	sICAM-1	463137.4 (398350.6, 520920)	418921.3 (375803.7, 458988.9)	**0.012***	30422.7	469008.3 (385456.1, 590741.4)	444406.2 (360751.2, 496985.4)	**0.0056****	45113.8
	sVCAM-1	463842.8 (400746.2, 583659)	434797.5 (383524.3, 520150.9)	**0.00024*****	37318	488951.9 (419294.6, 577680)	428840.4 (397779.4, 498670.5)	**0.0056****	50596.9
	TGF-b1	14105.1 (11680, 18298)	15167.4 (12051.4, 17848)	0.66	-352.2	15413 (12130.8, 20174.2)	17062.6 (12877, 21874)	0.38	-993.2
	TGF-b2	401.1 (362.1, 440.3)	388.7 (357.8, 428.1)	**0.012***	11.4	407.9 (366.9, 444.8)	403.9 (359.9, 443.1)	0.73	2.6
	TGF-b3	4.7 (3.5, 5.5)	3.8 (3.2, 4.9)	**0.013***	0.5	3.7 (3, 4.7)	3.6 (2.8, 4.8)	0.36	0.3
	TRAIL	173.2 (130, 197.6)	170.1 (142.5, 196.1)	0.82	1.1	167.1 (121.7, 215.5)	154.4 (121, 179.1)	0.41	7.6
	VEGF-A	44.2 (31, 60.7)	49.7 (35.7, 67.2)	0.31	-3.3	45.9 (35.6, 66)	51.9 (38.8, 66.8)	0.41	-2.7

a. Time effects were assessed by paired Mann–Whitney *U* test and adjusted by FDR.

t1=baseline, t2=9 weeks; IQR=interquartile range; * FDR-adjusted $p<0.05$, **FDR-adjusted $p<0.01$, *** FDR-adjusted $p<0.001$.

Table S5 *Time effects over 9 weeks on SCFAs.*

		Placebo				Synbiotic 2000			
**Age group**	**Analyte [µM]**	**t1 median (IQR)**	**t2 median (IQR)**	**FDR-adjusted p_{time}**	**Estimate of t1-t2**	**t1 median (IQR)**	**t2 median (IQR)**	**FDR-adjusted p_{time}**	**Esimate of t1-t2**
**Child**	Formic acid	188 (150.1, 401.7)	143.2 (111.9, 239.4)	0.36	25.761	203.6 (151.4, 319.6)	194.9 (130.1, 264.6)	0.89	6.812
	Acetic acid	5.2 (4, 32)	4.6 (3.3, 18.7)	0.36	1.914	9.5 (5.3, 24.7)	12.9 (6.5, 24.8)	0.87	-2.561
	Propionic acid	1.4 (0.9, 3.6)	1.3 (0.9, 2)	0.60	0.115	1.7 (0.9, 4.3)	2 (1, 3.9)	0.89	-0.041
	Butyric acid								
	Succinic acid	0.5 (0.4, 0.6)	0.6 (0.3, 0.8)	0.93	0.015	0.4 (0.2, 0.6)	0.4 (0.3, 0.6)	0.87	-0.043
	Isovaleric acid	0.4 (0.2, 0.5)	0.2 (0.2, 0.4)	0.60	0.057	0.2 (0.1, 0.3)	0.2 (0.1, 0.5)	0.95	0.002
**Adult**	Formic acid	438.1 (333.2, 488.3)	418.6 (326.5, 537.4)	0.12	-42.469	378.9 (317.1, 490.6)	399 (310, 481.9)	0.32	-16.1
	Acetic acid	25.3 (18.4, 34)	26.8 (16.7, 37.9)	0.17	-2.573	20 (12.4, 34.6)	25 (16.1, 38.2)	0.22	-4.5
	Propionic acid	3.3 (1.8, 4.6)	3.2 (2.1, 4.4)	0.17	-0.219	2.3 (1.5, 3.5)	2.2 (1.8, 3.8)	0.22	
	Butyric acid	0.4 (0.3, 0.6)	0.5 (0.3, 0.9)	0.087	-0.097	0.4 (0.3, 0.7)	0.5 (0.4, 0.8)	0.23	-0.34
	Succinic acid	3.1 (2.4, 3.6)	3.3 (2.5, 4.1)	0.17	-0.167	2.9 (2.5, 3.7)	3 (2.5, 3.9)	0.92	-0.006
	Isovaleric acid	0.2 (0.1, 0.4)	0.3 (0.1, 0.4)	0.12	-0.053	0.3 (0.1, 0.5)	0.4 (0.2, 0.5)	0.92	-0.001

a. Time effects were assessed by paired Mann–Whitney *U* test and adjusted by FDR.
t1=baseline, t2=9 weeks; IQR=interquartile range; * FDR-adjusted p<0.05, **FDR-adjusted p<0.01, *** FDR-adjusted p<0.001.
